# Immune stimuli shape the small non-coding transcriptome of extracellular vesicles released by dendritic cells

**DOI:** 10.1007/s00018-018-2842-8

**Published:** 2018-05-28

**Authors:** Tom A. P. Driedonks, Susanne G. van der Grein, Yavuz Ariyurek, Henk P. J. Buermans, Henrike Jekel, Franklin W. N. Chow, Marca H. M. Wauben, Amy H. Buck, Peter A. C. ‘t Hoen, Esther N. M. Nolte-‘t Hoen

**Affiliations:** 1 Department of Biochemistry and Cell Biology, Faculty of Veterinary Medicine, Utrecht University, Utrecht, The Netherlands; 2 Department of Human Genetics, Leiden University Medical Center, Leiden, The Netherlands; 3 Leiden Genome Technology Center, Leiden University Medical Center, Leiden, The Netherlands; 4 School of Biological Sciences, Centre for Immunity, Infection and Evolution, Institute of Immunology and Infection Research, University of Edinburgh, Edinburgh, UK; 5 Centre for Biomolecular and Molecular Informatics, Radboud Institute for Molecular Life Sciences, Radboud University Medical Center Nijmegen, Nijmegen, The Netherlands

**Keywords:** Immune activation, Immune suppression, Small RNA sequencing, Biomarker, Extracellular RNA

## Abstract

The release and uptake of nano-sized extracellular vesicles (EV) is a highly conserved means of intercellular communication. The molecular composition of EV, and thereby their signaling function to target cells, is regulated by cellular activation and differentiation stimuli. EV are regarded as snapshots of cells and are, therefore, in the limelight as biomarkers for disease. Although research on EV-associated RNA has predominantly focused on microRNAs, the transcriptome of EV consists of multiple classes of small non-coding RNAs with potential gene-regulatory functions. It is not known whether environmental cues imposed on cells induce specific changes in a broad range of EV-associated RNA classes. Here, we investigated whether immune-activating or -suppressing stimuli imposed on primary dendritic cells affected the release of various small non-coding RNAs via EV. The small RNA transcriptomes of highly pure EV populations free from ribonucleoprotein particles were analyzed by RNA sequencing and RT-qPCR. Immune stimulus-specific changes were found in the miRNA, snoRNA, and Y-RNA content of EV from dendritic cells, whereas tRNA and snRNA levels were much less affected. Only part of the changes in EV-RNA content reflected changes in cellular RNA, which urges caution in interpreting EV as snapshots of cells. By comprehensive analysis of RNA obtained from highly purified EV, we demonstrate that multiple RNA classes contribute to genetic messages conveyed via EV. The identification of multiple RNA classes that display cell stimulation-dependent association with EV is the prelude to unraveling the function and biomarker potential of these EV-RNAs.

**Electronic supplementary material:**

The online version of this article (10.1007/s00018-018-2842-8) contains supplementary material, which is available to authorized users.

## Introduction

Extracellular vesicles (EV) released by cells are considered as important mediators of intercellular communication [1, 2]. These 50–200 nm-sized vesicles are released by a broad range of cells, and have been detected in a wide range of body fluids, such as milk, plasma, urine, and semen [1, 3]. The collective term ‘EV’ refers to a heterogeneous population of secreted vesicles that are formed via different pathways. Exosomes are formed as intraluminal vesicles (ILVs) inside multi-vesicular endosomes (MVE) and are released upon fusion of the MVE with the plasma membrane, whereas microvesicles directly bud off from the plasma membrane [1]. These vesicle populations overlap in size and molecular composition, which currently hampers their discrimination based on biophysical or biochemical parameters [4–6].

EV are multi-component entities that transfer proteins, lipids, and RNA between cells. The RNA associated with EV mainly consists of small non-coding RNA species (ncRNA), which are thought to be of major importance in altering molecular processes in the recipient cell [3, 4, 7, 8]. In addition, the RNA composition of EV can give information about the (patho)physiological status of the EV-producing cell. Since EV can be easily obtained from body fluids, such EV-associated RNAs may be used as non-invasive biomarkers for the early detection of disease [9, 10]. The molecular composition of EV is often assumed to be a ‘snapshot’ of the producing cell, from which the tissue type or mutational status of the parental cell can be deduced [9, 11, 12].

Although the EV-RNA field receives major attention, with quickly rising numbers of publications on this topic, the technical difficulties to obtain pure EV remain underexposed. It is important to note that a substantial amount of extracellular RNA is not contained in EV, but is associated with ribonucleoprotein particles (RNPs), including argonaute 2 (AGO2), or lipoprotein complexes [13–15]. These contaminating structures have been shown to co-isolate together with EV via commonly used isolation procedures, and may, therefore, greatly affect the outcomes of experiments [4, 5, 16]. The currently most reliable method to obtain pure EV is by high-speed ultracentrifugation followed by density centrifugation in which EV are separated from contaminating structures based on differences in buoyant density [16]. Subsequently, analyzing the presence of common EV markers (such as CD9, CD63, and CD81) and absence of contaminant proteins should be performed to assess the purity of EV preparations used in the study [17, 18].

MiRNAs are the most intensely studied type of small RNA in EV, likely due to the interest in gene-regulatory functions of these RNAs [19–21]. It has been shown that changes in the activation status of the EV-producing cell lead to changes in the EV-miRNA composition [22–24]. Moreover, miRNAs in EV were shown to be functionally transferred between cells, leading to repression of genes in target cells [25, 26]. It is, therefore, thought that differences in the miRNA content of EV underlie distinct functional effects of EV released by differentially stimulated cells [22–24]. In addition, changes in EV-miRNA levels in plasma or serum have been associated with diseases such as cancer, rheumatoid arthritis, and Alzheimer’s disease [27–29].

Despite the interest in miRNAs, a larger part of EV-associated RNA consists of other ncRNA classes. We and others previously showed that EV released by cultured cells and EV present in a variety of body fluids contain many other small non-coding RNA species (20–300 nt), such as tRNA, snoRNA, snRNA, Y-RNA, Vault RNA, and SRP-RNA (also named 7SL) [23, 30–35]. Some of these non-coding RNAs are relatively enriched in EV, suggesting that these RNAs are specifically shuttled into EV for release into the extracellular milieu. Inside cells, the above-mentioned non-coding RNAs are known to play a role in basic cellular processes such as translation, RNA splicing, and RNA quality surveillance. Recent studies showed that full-length and fragmented forms of these non-coding RNAs can additionally be involved in other processes including immunological signaling, gene regulation, and guiding of other RNAs and nucleases [36–41]. However, it is not known whether activation stimuli imposed on the EV-producing cell affect the levels of these ncRNA classes in EV, similar to EV-associated miRNAs. Evaluation of cell stimulation-dependent changes in EV-associated ncRNA classes is a first step in uncovering their function in EV-mediated signaling processes and their potential as biomarkers indicative of the activation status of cells.

Here, we studied how changes in the activation/differentiation status of the parental cell affect the small non-coding RNA content of EV by employing a primary immune cell model, two different types of cell stimulation, and methods yielding highly pure EV populations. For our analysis, we used primary dendritic cells (DC), which are master regulators of immune responses and have been shown to communicate with various other immune cells via EV [2, 8, 42]. In addition, the use of DC-derived EV has been proposed as a strategy for cancer immune(chemo)therapy [43–46]. Depending on external stimuli, DC can be differentiated into functionally different phenotypes that either activate or down-regulate immune responses [47]. These DC, therefore, present a suitable model to investigate how different external stimuli affect incorporation of a broad range of RNA classes into EV. We compared cellular and EV-associated RNA levels in control, immune-stimulating, or immune-suppressing DC conditions using RNA deep sequencing and RT-qPCR. Our data indicate that differentiation signals imposed on dendritic cells affect the EV-associated levels of particular RNA classes, such as miRNA, snoRNA, and Y-RNA, while other non-coding RNA types remain largely unchanged. Moreover, only a minor part of the stimulation-induced changes in EV-RNA content reflects changes in cellular RNA levels. This study exemplifies how comprehensive analysis of RNA obtained from highly purified EV yields candidate small ncRNAs beyond miRNAs for further exploration as biomarkers or functional entities within EV.

## Materials and methods

### Cell culture

Complete culture medium was prepared by supplementing Iscove’s Modified Dulbecco’s Medium (IMDM, Lonza, Verviers, Belgium) with 2 mM Ultraglutamine (Lonza, Verviers, Belgium), 10% fetal calf serum (FCS, GE Healthcare Bio-Sciences, Pasching, Austria), 100 IU/ml penicillin and 100 μg/ml streptomycin (Gibco, Paisley, United Kingdom), and 50 µM β-mercaptoethanol. The GM-CSF producing NIH 3T3 cell line (R1) was grown in complete medium. Primary bone marrow cells were grown in complete medium supplemented with 30% conditioned medium from R1 cells. To prepare EV-depleted medium, a mixture of 150 ml conditioned R1 culture supernatant and 42.5 ml FCS (end concentration 30% FCS) was depleted of EV by overnight centrifugation at 100,000*g* in an SW28 rotor (*k*-factor 334.2) (Beckman Coulter, Brea, CA). The EV-depleted supernatant was carefully pipetted from the tubes, leaving 5 ml in the tubes to prevent disturbance of the pellet. This supernatant was filtered through a 0.22 μm bottle top filter (Millipore, Billerica, MA) after which additional IMDM, ultraglutamine, antibiotics, and β-mercaptoethanol were added to prepare complete medium as indicated above. The efficiency of EV depletion from culture medium, as regularly assessed by high-resolution flow cytometric analysis, is on average ~ 90%. Primary bone marrow cells were flushed from the femur and tibia of 8–12-week-old C57bl/6 mice and differentiated to dendritic cells according to Lutz et al. [48]. To generate tolerogenic DC, cells were treated from day 2 onward with 10 nM 1α,25-dihydroxyvitaminD3 (Sigma, St Louis, MO). To generate immunogenic DC, 1 µg/ml lipopolysaccharide (LPS, cat. L-2630, Sigma, St. Louis, MO) was added on day 12. On day 13, non-adherent and semi-adherent cells were recovered and cultured at 3 × 10^6^ cells/dish for 20 h in EV-depleted culture medium. Cell viability, as determined by Trypan blue exclusion, did not differ between treatments and was above 90% for all cultures. All cells were maintained at 37 °C and 5% CO_2_ in a humidified incubator. Experiments were approved by the institutional ethical animal committee at Utrecht University (Utrecht, The Netherlands).

### Flow cytometry

Day 12 bone marrow DC (BMDC) were collected and labeled for 30 min in PBS + 1% BSA (Bovine Serum Albumin, cat. K45-001, GE Healthcare Bio-Sciences, Pasching, Austria) containing anti-CD11c-APC (eBioscience, clone N418, 1:400), anti-MHCII-FITC (eBioscience, clone M5/114.15.2, 1:1500), and anti-CD40-PE (BD Biosciences, clone 3/23, 1:200), or anti-CD86-PE (eBioscience, clone GL1, 1:200) or anti-CD274-PE (PD-L1, eBioscience, clone M1H5, 1:400). As control, corresponding isotype control antibodies (eBioscience, San Diego, CA) were used. Surface labeling was analyzed using a FACS Calibur Flow Cytometer (BD Biosciences, San Jose, CA). Data analysis was performed using FCS Express V3 (DeNovo Software, Glendale, CA).

### Fluorescent labeling and purification of EV

Conditioned cell supernatants were pooled to volumes of 100–140 ml per condition and were subjected to differential centrifugation as described previously [49]. In brief: supernatant was sequentially centrifuged 2 × 200*g* for 10 min, 2 × 500*g* for 10 min, and 1 × 10,000*g* for 30 min. Next, EV were pelleted by ultracentrifugation at 100,000*g* for 65 min using an SW28 rotor (*k*-factor 334.2) (Beckman Coulter, Brea, CA). For EV quantification by high-resolution flow cytometry, pellets were resuspended in 20 µl PBS + 0.2% BSA (cleared from aggregates by overnight ultracentrifugation at 100,000*g*) and labeled with 1.5 µl PKH67 (Sigma, St. Louis, MO) in 100 µl Diluent C per pellet. For EV-RNA isolation, 100,000 g pellets were resuspended in 50 µl PBS + 0.2% BSA. PKH67-labeled samples or samples for RNA isolation were mixed with 1.5 ml 2.5 M sucrose, and overlaid with a linear sucrose gradient (2.0–0.4 M sucrose in PBS). Gradients were centrifuged 15–18 h at 192,000*g* in a SW40 rotor (*k*-factor 144.5) (Beckman Coulter, Brea, CA). PKH67-labeled EV were used for high-resolution flow cytometric analysis (see below). Alternatively, the fractions with densities of 1.12–1.18 g/ml (as measured by refractometry) were pooled, diluted six times in PBS + 0.2% EV-depleted BSA, and centrifuged again at 192,000*g* for 65 min in a SW40 rotor (*k*-factor 144.5) prior to EV-RNA isolation.

### RNA isolation

Small RNA was isolated from EV pellets and from 1 × 10^6^ cells using the miRNeasy Micro kit according to the small RNA enrichment protocol provided by the manufacturer (Qiagen, Hilden, Germany). RNA yield and size profile were assessed using the Agilent 2100 Bioanalyzer with Pico 6000 RNA chips (Agilent Technologies, Waldbronn, Germany).

### Preparation of small RNA sequencing libraries

50 ng cellular small RNA and 2 ng EV-derived small RNA was treated with DNase [Turbo DNA-free kit (Life Technologies, Carlsbad, CA)] according to the manufacturer’s instructions. The DNase-treated RNA was pelleted using Pellet Paint (Merck, Darmstadt, Germany) according to the manufacturer’s instructions, reconstituted in milliQ (MQ) and subsequently subjected to ribosomal RNA depletion using RiboZero Gold kit (Human/Mouse/Rat) (Illumina, San Diego, CA) according to the manufacturer’s instructions. This is required to deplete residual rRNA from cellular RNA samples and, for comparability, we subjected EV-RNA samples to the same procedure. Hereafter, RNA was pelleted with Pellet Paint and reconstituted into 6 µl MQ. cDNA libraries were prepared using the NebNext smallRNA library prep kit for Illumina (New England Biolabs, Ipswich, MA), according to the manufacturer’s instructions but with the following adaptations: 3′ adapter-ligation was carried out overnight at 16 °C; PCR amplification was done using Kapa HiFi Readymix 2× PCR mastermix (Kapa Biosystems, Wilmington, MA) using barcoded primers and the following PCR programme: 2 min at 95 °C, 15 cycles of 20 s at 98 °C, 30 s at 62 °C, 15 s at 70 °C, and a final elongation step of 5 min at 70 °C. cDNA was purified using magnetic AMPure XP beads (Beckman Coulter, Brea, CA) and quantified using Agilent 2100 Bioanalyzer and DNA HiSensitivity chips (Agilent Technologies, Waldbronn, Germany). Adapter dimers (126 nt in size) were removed by running the cDNA on 6% TBE gels (Life Technologies, Carlsbad, CA) for 60 min at 145 V, after which cDNA products of 15–300 nt (+ 126 nt adapters) were cut from the gel and purified. Subsequently, all libraries were pooled at equimolar ratios and run on a 4–12% TBE gel (Life Technologies, Carlsbad, CA). Size fractions of 15–25 nt (includes miRNAs), 25–60 nt, 60–80 nt (includes tRNAs), and 80–275 nt (each + 126 nt adapters) were cut from the gel, and purified and pooled in a volumetric ratio of 2:2:3:3. Sequencing was done using 150 bp paired-end reads on an Illumina HiSeq 4000 machine (Illumina, San Diego, CA) at ServiceXS (Leiden, The Netherlands).

### RNAseq data analysis

All data was processed as paired-end reads. Data quality was checked with FastQC and reads were processed with cutadapt (version 1.8) to remove low-quality reads, clip the reads to 100 bp, and remove adapter sequences. Sequences with a minimal length of 17 bp after adapter trimming were retained to ensure high quality reads (cutadapt -q 30 -u -51 -U -51 -n1 --minimum-length 17 -a AGATCGGAAGAGCACACGTCTGAAC -A GATCGTCGGACTGTAGAACTCTGAA). Reads were mapped to the mouse genome mm10 using gsnap (v2014-12-23; gsnap --novelsplicing 1 --npaths 3 --format sam). Count tables were prepared using Htseq (v0.6.1p1; htseq-count --minaqual 0 --format bam --order pos --stranded yes). For miRNA count tables, miRbase v20 annotation was used. Gene annotation for snoRNA, snRNA, and miscRNA was retrieved from Biomart (Ensembl 84). tRNA and rRNA chromosomal positions were retrieved from the UCSC mm10 repeat mask track. Reproducibility of triplicate experiments (performed on different days) was assessed by evaluating Pearson correlation values (after logarithmic transformation of the small RNA counts).

Differential abundance was determined separately for each ncRNA class, and separately for EV and cells, using the edgeR package in R [50]. Data were normalized using the TMM method (weighted trimmed mean of M values). Estimating the common, trended (overexpression values), and tagwise dispersion, a generalized linear model, containing the effect of cell stimulation and the effect of the day-to-day variation between triplicate experiments, was fit. The log-likelihood ratio test was used to evaluate differential expression between treatments relative to control. *p* values were adjusted for multiple testing using Benjamini and Hochberg’s false discovery rate (FDR). Average fold-change over three independent experiments and standard deviation were plotted. Analysis of RNA fragments was done using the UCSC genome browser and Integrated Genome Viewer [51].

### Quantitative real-time PCR

cDNA was generated from cellular or EV-derived small RNA using the miScript RT2 kit (Qiagen, Hilden, Germany). An equivalent of 20 pg RNA was used per qPCR reaction and mixed with 100 nM primers (Isogen Life Sciences, De Meern, The Netherlands) and 4 µl SYBR Green Sensimix (Bioline Reagents Ltd., United Kingdom) in an 8 µl reaction. No-RT-controls confirmed the absence of genomic DNA and non-specific amplification.

Cycling conditions were 95 °C for 10 min followed by 50 cycles of 95 °C for 10 s, 57 °C for 30 s, and 72 °C for 20 s. All PCR reactions were performed on the Bio-Rad iQ5 Multicolor Real-Time PCR Detection System (Bio-Rad, Hercules, CA). Quantification cycle (Cq) values were determined using Bio-Rad CFX software using automatic baseline settings. Thresholds were set in the linear phase of the amplification curve.

### High-resolution flow cytometric analysis of EV

High-resolution flow cytometric analysis of PKH67-labeled EV was performed using a BD Influx flow cytometer (BD Biosciences, San Jose, CA) with an optimized configuration, as previously described [49, 52]. In brief, we applied threshold triggering on fluorescence derived from PKH67-labeled EV passing the first laser. Forward scatter (FSC) was detected with a collection angle of 15°–25° (reduced wide-angle FSC). Fluorescent 100- and 200-nm polystyrene beads (FluoSpheres, Invitrogen, Carlsbad, CA) were used to calibrate the fluorescence and rw-FSC settings. Sucrose gradient fractions containing PKH67-labeled EV were diluted 25× in PBS and vortexed just before measurement. This dilution factor was sufficient to avoid ‘coincidence’ (multiple EV arriving at the measuring spot at the same time), thereby allowing accurate quantitative comparison of EV numbers in different conditions. Moreover, samples were measured at maximally 10,000 events per second, which is far below the limit in the electronic pulse processing speed of the BD Influx [53].

### Western blotting

Cell pellets were lyzed in PBS + 1% Nonidet-P40 with protein inhibitor cocktail (Roche, Basel, Switzerland) for 15 min on ice. Nuclei were spun down at 16,000 g for 15 min at 4 °C, supernatant was used for Western blotting. Cell lysates and EV were denatured in SDS-sample buffer at 100 °C for 3 min, and separated using 12% SDS-PAGE gels, after which proteins were transferred onto Immobilon-P 0.45 μm PVDF membranes (Millipore, Cork, Ireland). After blocking for 1–2 h in blocking buffer (0.5% Cold Fish Skin Gelatin (Sigma-Aldrich, St. Louis, CA) in PBS + 0.05% Tween-20), blots were incubated overnight at 4 °C with primary antibodies [anti-mouse-CD9 (eBioscience, clone KMC8, 1: 1000), anti-mouse-CD63 (MBL, clone D263-3, 1:1000), anti-mouse–galectin-3 (eBioscience, clone M3/38, 1:500), anti-MHCII-p55 (GenScript, Piscataway, NJ, custom Ab raised against MHCII bèta chain peptide sequence RSQKGPRGPPPAGLLQC, 1:5000), or anti-mouse-beta-actin (ThermoScientific, polyclonal PA1-16889, 1:5000)] in blocking buffer, and washed and incubated for 1–2 h with HRP-coupled secondary antibodies (Dako, cat P0450 and P0448, 1:5000). ECL solution (ThermoScientific, SuperSignal West Dura Extended Duration Substrate, cat. 34075) was used for detection on a Chemidoc imager (Bio-Rad, Hercules, CA). Images were analyzed by the Image Lab software (Bio-Rad, Hercules, CA).

### Nanoparticle tracking analysis (NTA)

EV purified by density gradient ultracentrifugation as described above were resuspended in 50 µl PBS + 0.4% EV-depleted BSA. Samples were diluted 20 times in PBS (confirmed to be particle-free when analyzed with the same settings as used for the EV samples) before measurement on a Nanosight NS500 instrument (Malvern, Worchestershire, UK). Data acquisition and processing were performed using NTA software 3.1. Each sample was recorded for 5 times 30 s, 25 frames per second, camera level 14, detection threshold 5.

### Electron microscopy

EV and RNP were separated by density gradient centrifugation as described above, and resuspended in 15 µl PBS. 3 µl aliquots of either EV or RNP were added on top of carbon-coated 300 mesh, copper grids, and incubated for 2 min at room temperature. The grids were then washed two times in 50 µl PBS and stained with 2% uranyl acetate. Imaging was performed on a T20 electron microscope (FEI) operated at 200 keV. Images were recorded on a CCD Eagle camera (FEI).

### Northern blotting

Cellular or EV-derived small RNA (between 100 ng and 1 ng per lane, as indicated) was denatured in gel loading buffer II (Invitrogen) for 2 min at 70 °C and snap-cooled on ice before loading on a denaturing 15% PAGE gel (National Diagnostics, Nottingham, UK) as described previously [54]. RNA was transferred onto a Hybond-N Nylon membrane (Amersham Pharmacia Biotech, GE Healthcare Bio-Sciences, Pasching, Austria) and chemically crosslinked with 1-ethyl-3-(3-dimethylaminopropyl) carbodimide (Sigma) at 55 °C for 2 h [55]. ^32^P-labeled DNA oligo probes perfectly complementary to each RNA species were hybridized overnight at 42 °C in PerfectHyb (Sigma) solution. Blots were analyzed by phosphorimaging using a Typhoon Scanner (GE Healthcare).

### List of DNA oligo probes

#### RT-qPCR probes

The following forward primers were used in RT-qPCR (5′ to 3′) in combination with the miScript universal reverse primer.

miR-146a-5p: TGA GAA CTG AAT TCC ATG GGT
miR-155-5p: GGG TTA ATG CTA ATT GTG ATA G
miR-9-5p: GGG TCT TTG GTT ATC TAG C
miR-10a-5p: TAC CCT GTA GAT CCG AAT TTG TG
miR-27b-3p: TTC ACA GTG GCT AAG TTC TGC
miR-378-5p: ACT GGA CTT GGA GTC AGA AG
mY1: GTT ATC TCA ATT GAT TGT TCA CAG TC
mY3: GGC TGG TCC GAG TGC AGT GG
RNU1: CCATGATCACGAAGGTGGTTT
RNU6: CTCGCTTCGGCAGCACA
snord65: TAGTGGTGAGCCTATGGTTTT
snord68: AGTACTTTTGAACCCTTTTCCA

#### Northern oligo’s

Y1_5p: TTG AGA TAA CTC ACT ACC TTC GGA CCA GCC
Y1_3p: GTC AAG TGC AGT AGT GAG AAG
5′-tRNA-Glu_CTC: CCG AAT CCT AAC CAC TAG ACC ACC AG
5′-tRNA-Gly_GTT: GCA TTG GTG GTT CAG TGG TAG AAT TCT CGC C

### Statistical analyses

Differences between the numbers of EV released by LPS- and VitD3-stimulated DC versus control DC were analyzed by one-way ANOVA with Dunnett’s two-sided post hoc test. Similar statistical testing was performed on differences in RNA yield. Differences in RNA biotypes in EV and cells were analyzed by two-tailed *t* test. Differences in fold-changes of individual RNAs as measured in RT-qPCR were analyzed by one-way ANOVA. Significance was defined as *p* < 0.05. Statistical tests on data not derived from RNA sequencing were done in SPSS (v24, IBM).

### Availability of data and materials

Raw data and count tables are deposited in the GEO database under accession number GSE105151. Written details on experimental procedures have been submitted to the EV-TRACK knowledgebase (EV-TRACK ID: EV170030) [5].

## Results

**Fig. 1:**
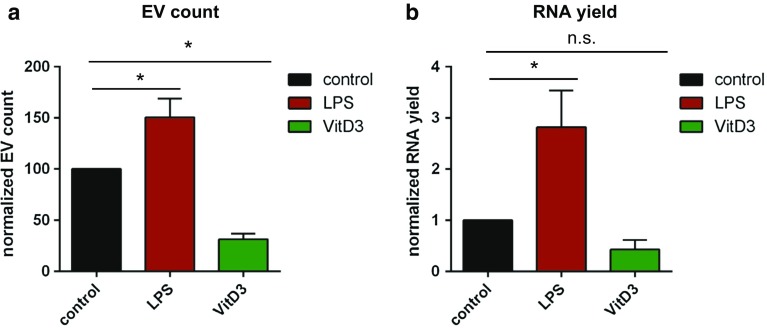
LPS- and VitD3-stimulated DCs release different numbers of extracellular vesicles. **a** EV derived from equal numbers of control, and LPS- and VitD3-treated DC were fluorescently labeled using PKH67, separated from RNPs and free dye by sucrose density centrifugation, and quantified by high-resolution flow cytometry. Indicated are the number of fluorescent events detected in 30 s, normalized to the control condition (mean ± SD of *n* = 3 independent experiments, one-way ANOVA with Dunnett’s two-sided post hoc test, *p < 0.05. **b** Bioanalyzer-based quantification of RNA isolated from EV derived from equal numbers of cells purified by density gradient centrifugation. Small RNA yields were normalized to the control DC condition. Indicated are the mean ± SD values of *n* = 4 independent experiments (one-way ANOVA with Dunnett’s two-sided post hoc test, *p < 0.05)

Functionally distinct subsets of mouse bone marrow-derived DCs were generated by exposure of cells to LPS, thereby inducing immune-stimulatory DC (LPS-DC), or to 1α,25-dihydroxyvitaminD3, thereby inducing immune-suppressive DC (VitD3-DC) [56]. Untreated DC were used as control cells. The different DC subtypes displayed the expected differences in expression of the activation markers, with LPS-DC showing increased levels of MHC class II and CD86 expression, while VitD3-DC showed increased PD-L1/CD86 ratios [57] and resistance to LPS activation [58] (Supplementary Fig. 1A–F). We isolated EV from cell culture supernatant of LPS-DC, VitD3-DC, and control DC using differential (ultra)centrifugation followed by density gradient separation, as previously published by our group (Supplementary Fig. 2A and [52]). Transmission electron microscopic analysis indicated that low-density fractions contained 100–150 nm-sized EV, whereas no EV were observed in high-density fractions enriched in protein complexes (Supplementary Fig. 2B). Our in-house developed high-resolution flow cytometric method was used for high-throughput EV analysis at the single particle level [49, 52]. The light scatter patterns induced by EV from differently stimulated DC were highly similar (Supplementary Fig. 2C). However, we detected differences in the number of released EV, with LPS-DC releasing more, and VitD3-DC releasing less EV than non-treated control DC (Fig. 1a). These quantitative differences were confirmed by nanoparticle tracking analysis (NTA) (Supplementary Fig. 2D). In addition, the NTA data indicated that the majority of EV observed in all conditions were in the 100–200 nm size range. Western blot analysis showed that the DC-derived EV contained variable levels of common EV proteins such as CD9, MHCII, CD63, and Galectin-3 [59], whereas abundant cellular proteins such as beta-actin were not detected (Supplementary Fig. 2E). These data demonstrate that VitD3 and LPS treatment of DC differentially affect the number and protein content of EV released by these cells.

**Fig. 2:**
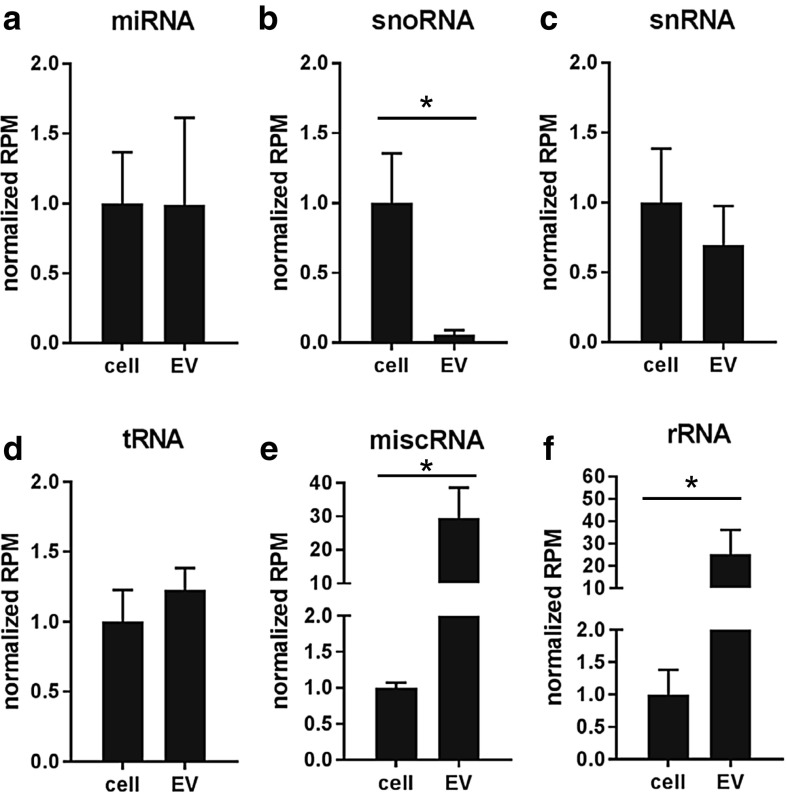
Cells and EV differ in the relative abundance of different small RNA classes. **a**–**f** The relative abundance of the indicated ncRNA classes in cells versus EV was assessed by small RNAseq analysis of cellular and EV-associated RNA. Total read count for each RNA class was normalized to total library size. To compare the distribution of ncRNA classes between cells and EV, normalized RNA class read counts in EV were scaled to the normalized RNA class read counts in cellular RNA (set to 1). Indicated are mean values ± SD of *n* = 3 independent experiments (two-tailed *t* test on normalized CPM values, *p < 0.05)

Next, we analyzed how LPS- or VitD3 treatment of DC affected the RNA content of EV released by these cells. It is important to note that extracellular RNA can be associated with either EV or ribonucleoprotein complexes, which may sediment at similar centrifugal force [4]. Using density gradient-based purification, we separated EV from contaminating ribonucleoprotein complexes. Depending on the differentiation status of the parental DC, the EV-free ribonucleoprotein fraction contained between 26 and 55% of total extracellular RNA released from cells (Supplementary Fig. 2F–G). The total amount of EV-associated RNA released by equal numbers of control, LPS-, and VitD3-treated DC was different and reflected the differences in EV numbers (Fig. 1a, b). To assess whether DC stimulation altered the RNA composition of EV, we prepared sequencing libraries of EV-associated and cellular small RNA from control-, LPS-, and VitD3-DC cultures (*n* = 3 biological replicates). Sequencing libraries were prepared using an adapter-ligation-based method routinely used in miRNA profiling [60]. Such a method has been frequently applied in studies to demonstrate the presence of miRNA and other small non-coding RNA types in EV (e.g., [23, 24, 31, 32, 61]). All libraries contained RNAs with lengths ranging between 20 and 300 nucleotides. Since short RNA molecules have a selective advantage during PCR amplification and pre-amplification on the sequencing flow-cell, leading to an apparent over-representation of short read lengths, we enriched the libraries for longer RNAs to ensure sufficient coverage of these sequences. To this end, cellular and EV-associated cDNA libraries were split into different size fractions of 15–25 nt (includes miRNAs), 25–60 nt, 60–80 nt (includes tRNAs), and 80–275 nt. Subsequently, we enriched for longer RNA molecules by pooling these size fractions in a volumetric ratio of 2:2:3:3 (for details, see “Materials and methods”). This, indeed, resulted in a better coverage of 100–300 nt mid-size RNAs, such as full-length Vault RNA (142 nt) (Supplementary Fig. 3). More than 13 million reads were obtained for each library (Supplementary Table 1). Pearson coefficients for biological replicate samples were ≥ 0.85 for EV, and ≥ 0.95 for cells (Supplementary Fig. 4). As expected based on the previous studies [23, 30–32, 34], we detected the presence of various small RNA classes in EV (miRNA, snoRNA, snRNA, tRNA, miscRNA, and rRNA). Some RNA types were enriched in EV, such as Y-RNA, Vault RNA, and 7SL RNA classified as ‘miscRNA’, while other RNA types, such as snoRNA, were relatively less abundant in EV compared to cells (Fig. 2). Since we depleted ribosomal RNA before preparation of the sequencing libraries, apparent enrichment of rRNA in EV is probably caused by differences in depletion efficiency between cells and EV. MiRNA and snRNA abundance was similar between cells and EV. Stimulation of DC with LPS or VitD3 did not lead to major changes in the distribution of sequencing reads over different RNA classes in EV and cells (Supplementary Fig. 5A–B).

**Fig. 3:**
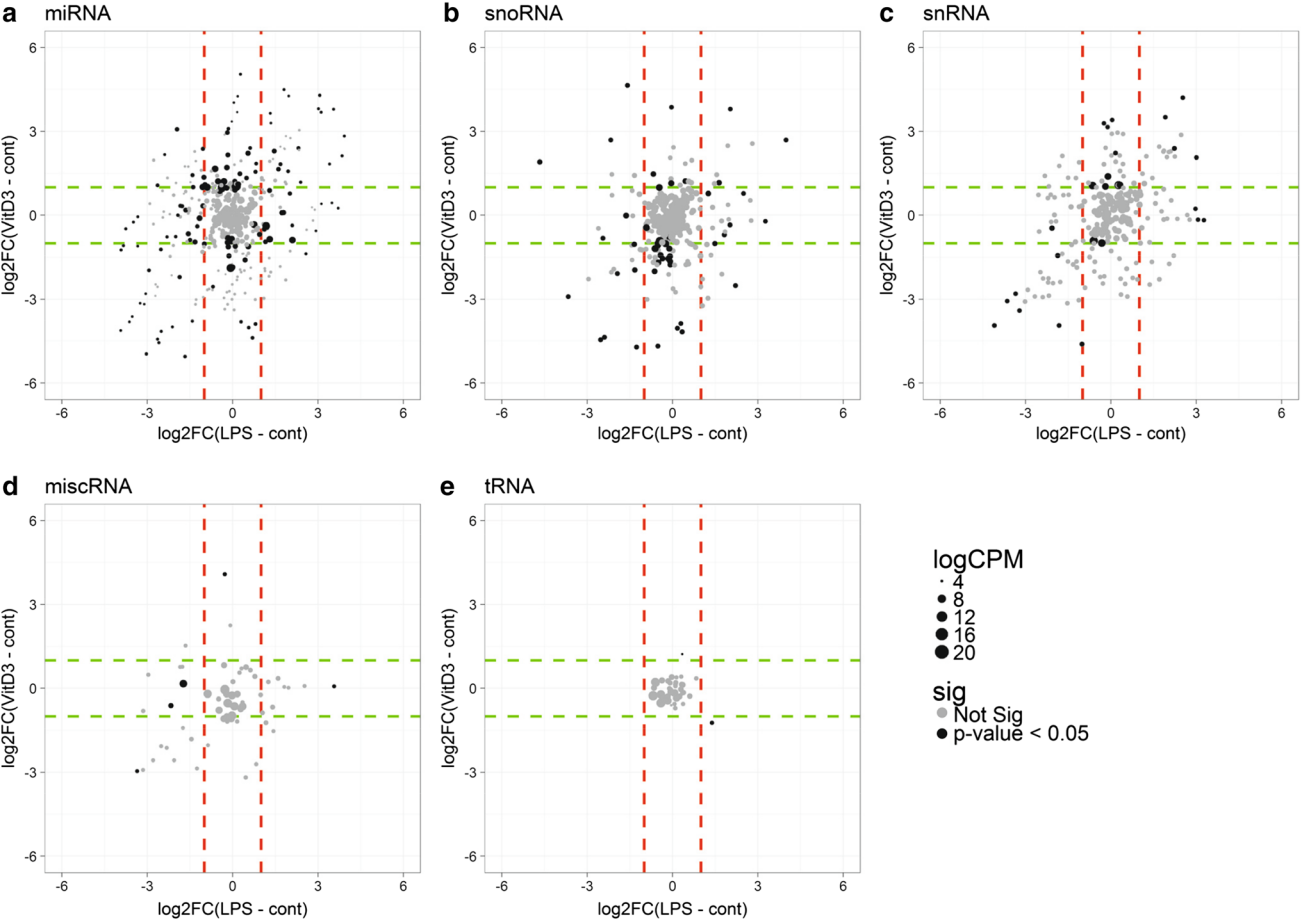
LPS- versus VitD3-induced changes in EV-associated RNA classes. EV-associated RNA from control, LPS, and VitD3 conditions was isolated and analyzed by RNA sequencing. Read counts for individual RNAs were normalized to the total read counts of each RNA class. **a**–**e** LPS- or VitD3-induced fold changes and corresponding *p* values were calculated relative to the control condition with edgeR GLM method. Data are expressed as log2fold change in LPS-EV relative to control-EV versus log2fold change in VitD3-EV relative to control-EV. Cutoffs for log2fold changes larger or smaller than 1 are indicated with dashed lines (red = LPS; green = VitD3), so all data points beyond these lines are differentially expressed with log2FC > 1. Dot size represents the normalized abundance (logCPM) of individual RNAs. RNAs that changed with non-adjusted *p* < 0.05 are highlighted in black

Next, we assessed whether, within each of the different classes, the levels of individual RNAs in EV changed as a result of cell stimulation. Hereto, read counts were normalized per RNA class, after which the fold changes in reads per million (RPM) of individual RNAs in LPS or VitD3 over control conditions were calculated. The scatter plots in Fig. 3a–e display the log-fold changes induced by LPS versus VitD3 treatment for the indicated RNA classes (dashed lines correspond to a log2fold change of 1). Dot size indicates the average (*n* = 3) abundance of a transcript in EV and RNAs with non-adjusted *p* < 0.05 are highlighted in black. The scatter plots of these data indicated that LPS- and VitD3-treatment affected the levels of miRNA, snoRNA, snRNA, and miscRNA in EV (Fig. 3a–e). Some of the stimulation-induced changes were similar for LPS and VitD3 conditions, while other RNAs show opposing alterations, or change only in one of the stimulation conditions.

**Fig. 4:**
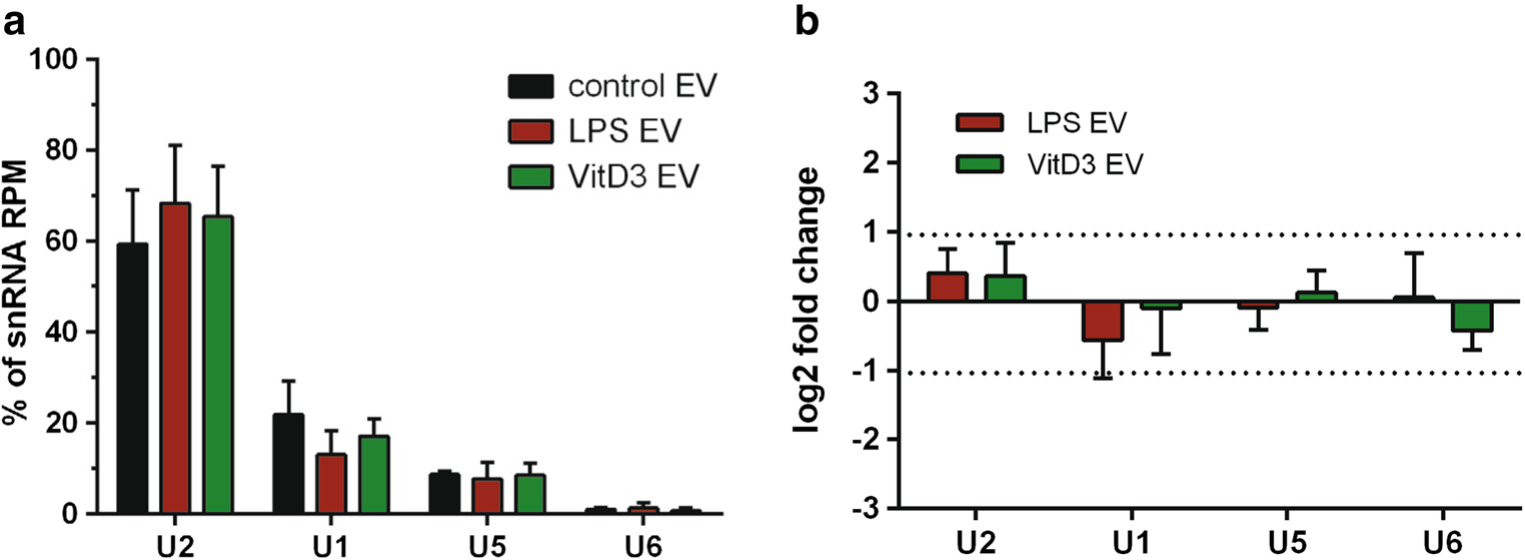
EV from LPS- and VitD3-stimulated DC display stable levels of abundant snRNAs. EV-associated RNA from control, LPS, and VitD3 conditions was isolated and analyzed by RNA sequencing. Read counts for individual RNAs were normalized to the total read counts of each RNA class. **a** Read counts for the top-four most abundant snRNAs, constituting 96% of snRNA reads in EV-RNA, are expressed as percentage of the total snRNA read count in EV. **b** Data from **a** are expressed as fold change in the indicated snRNA levels of VitD3- or LPS-EV relative to control-EV (mean ± SD of *n* = 3 experiments; in one-way ANOVA, no significant differences were observed)

Of the tested RNA classes, tRNA levels in EV showed the lowest rate of change due to LPS or VitD3 stimulation. 97% of tRNA reads mapped to six different tRNA isoacceptors (GluCTC, GlyGCC, LysCTT, GluTTC, GluCTG, and LysTTT) (Supplementary Fig. 6A). Although relatively high tRNA read counts were observed in the EV-associated RNA pool (Supplementary Fig. 5B), the levels of these six abundant tRNAs remained stable (log2FC < 1) in response to cellular stimulation (Supplementary Fig. 6B). Besides tRNAs, the highly purified EV populations used in this study also contained substantial levels of snRNA, which is an RNA type known to be mainly confined to the nucleus. Although cell stimulation seemed to induce changes in EV-associated snRNA levels (Fig. 3c), many of the individual data points mapped to multicopy genes with highly similar sequences (> 95% sequence identity) corresponding to known snRNAs. Cumulative analysis of these multicopy genes indicated that 96% of snRNA reads mapped to four different snRNAs (U1, U2, U5, and U6) (Fig. 4a), of which the levels in EV did not significantly differ between stimulation versus control conditions (Fig. 4b). Thus, the levels of snRNAs and tRNAs, which are commonly detected RNA types in EV, remained constant upon different immune stimuli imposed on the EV-producing cells.

In contrast to tRNAs and snRNAs, the EV-associated levels of three other RNA types tested here, i.e., miRNAs, snoRNAs, and miscRNAs, changed in response to the different stimulate imposed on the DC. We aimed to identify the RNAs differentially present in EV released by LPS- versus VitD3-treated DC, since such RNAs may underlie distinct effects of EV on recipient cells.

**Fig. 5:**
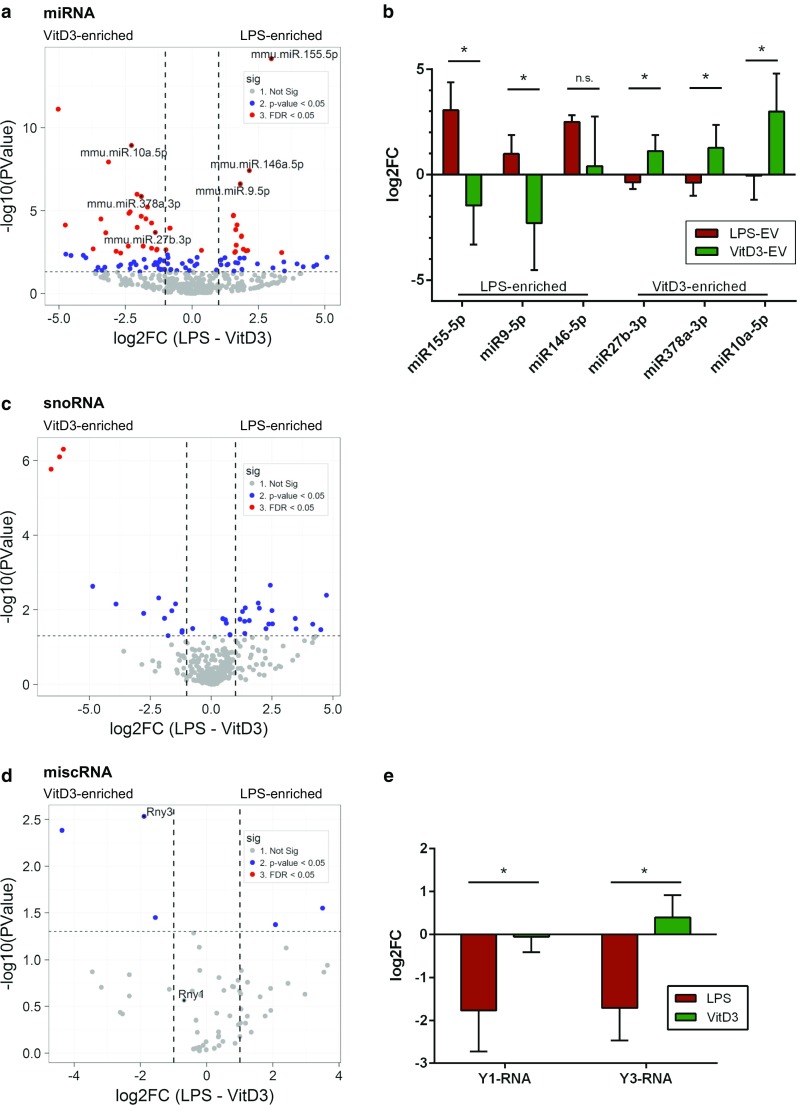
EV from LPS-/VitD3-stimulated DC display differences in the levels of several miRNAs, snoRNAs, and Y-RNAs. EV-associated RNA from control, LPS, and VitD3 conditions was isolated and analyzed by RNA sequencing. Read counts for individual RNAs were normalized to the total read counts of each RNA class. LPS- or VitD3-induced fold changes and corresponding *p* values were calculated relative to the control condition. **a** Volcano plots of EV-associated miRNAs in LPS versus VitD3 conditions. Thresholds for twofold change and non-adjusted *p* < 0.05 are indicated. Data points represent average values of *n* = 3 biological replicates. Significant changes are indicated with different colours. Grey: non-significant, blue: non-adjusted *p* value < 0.05, and red: FDR < 0.05. **b** RT-qPCR validation of six miRNAs showing differential abundance between LPS-EV and VitD3-EV. Data are expressed as log2fold change in LPS- and VitD3-EV compared to control-EV. Indicated are the mean ± SD values of *n* = 4 independent experiments, one-way ANOVA, *p < 0.05. Volcano plots of EV-associated snoRNAs (**c**) and miscRNAs (**d**) in LPS versus VitD3 conditions. Thresholds for twofold change and non-adjusted *p* < 0.05 are indicated. Data points represent average values of *n* = 3 biological replicates. Significant changes are indicated in different colours. Grey: non-significant, blue: non-adjusted *p* value < 0.05, red: FDR < 0.05. **e** RT-qPCR validation of Y3 and its family member Y1 showing differential abundance between LPS-EV and VitD3-EV compared to control-EV. Indicated are the mean ± SD values of *n* = 4 independent experiments, one-way ANOVA, *p < 0.05

Within the miRNA group, we observed the highest number of RNAs that differed between the LPS and VitD3 groups with a fold change > 2 and with FDR values < 0.05 (Fig. 5a, red dots). Interestingly, for the majority (15 out of 20) of the top miRNAs that differed significantly between VitD3 and LPS conditions with lowest FDR values, DC functions have been described previously (Table 1 and [62–73]).

**Table 1:** MicroRNAs enriched in LPS- or VitD3-EV with known functions in DC

Identifier	Enriched in	FDR	Function in DC	References
mmu-miR-155-5p	LPS-EV	4.04E−12	Master regulator in DC maturation	[62]
mmu-miR-708-3p	VitD3-EV	2.21E−09	Downregulated in mature/activated DC	[63]
mmu-miR-10a-5p	VitD3-EV	2.31E−07	Inhibits DC activation and Th1/Th17 cell immune responses	[64]
mmu-miR-146a-5p	LPS-EV	4.53E−06	Down regulates IL-12p70, IL-6, and TNF-α production by DC	[65]
mmu-miR-9-5p	LPS-EV	1.86E−05	Regulatory circuitry controlling monocyte activation by LPS	[66]
mmu-miR-223-5p	VitD3-EV	6.82E−05	Repression of pro-inflammatory cytokine release by DC	[67]
mmu-miR-378a-3p	VitD3-EV	8.24E−05	Upregulated in VitD3-treated DC	[68]
mmu-miR-203-3p	VitD3-EV	0.000328	Upregulated in tolerogenic DC	[69]
mmu-miR-199a-3p	VitD3-EV	0.000484	Upregulated in tolerogenic DC	[69]
mmu-miR-27b-5p	VitD3-EV	0.000578	Suppression of inflammatory cytokine production via NF-κB	[69]
mmu-miR-7a-5p	LPS-EV	0.000703	Upregulated in LPS/IFNg stimulated DC	[69]
mmu-miR-126a-3p	VitD3-EV	0.000946	Reduces the responsiveness of DCs to TLR7/9 ligands	[70]
mmu-miR-708-5p	VitD3-EV	0.000946	Suppresses NF-κB signaling	[71]
mmu-miR-181b-3p	VitD3-EV	0.001923	Inhibition of CD40 and MHCII expression	[72]
mmu-miR-27a-5p	VitD3-EV	0.002511	Suppression of inflammatory cytokine production	[73]

EV-associated RNA from control, LPS, and VitD3 conditions was isolated and analyzed by RNA sequencing. Read counts for individual RNAs were normalized to the total read counts of each RNA class. LPS- or VitD3-induced fold-changes and corresponding *p* values were calculated relative to the control condition with edgeR GLM method. We created a top 20 list of miRNAs with the lowest FDR values. Indicated are miRNAs from this top 20 for which DC-related functions have been reported

MiRNAs known to be involved in the pro-inflammatory function of DC were enriched in LPS-induced EV, whereas EV from VitD3-treated DC were enriched in miRNAs that dampen immune-stimulatory signaling cascades in DC. We validated three LPS-enriched and three VitD3-enriched RNAs by RT-qPCR. The use of stable reference transcripts in RT-qPCR analysis is important to ensure reliable normalization between different samples. However, the identity and general applicability of reference transcripts stably present in EV are understudied. Here, we used our sequencing data set to select four non-coding RNAs that were present in EV at comparable levels across all conditions, and confirmed their stability by RT-qPCR (Supplementary Fig. 7A–B). We normalized our qPCR data to the geometric mean of these four genes to minimize errors caused by technical and/or biological variation [74]. Using this normalization strategy, RT-qPCR analysis confirmed the sequencing data for the tested miRNAs, although miR146-5p did not reach significance (Fig. 5b). Together, these data indicate that opposing stimuli imposed on the same type of parental cell induce the release of functionally different sets of miRNAs via EV.

Besides changes in miRNA levels, the levels of several EV-associated snoRNAs were found to differ between LPS and VitD3 conditions (Fig. 5c), with three H/ACA box snoRNAs (snora2b, snora32, and snora55) displaying FDR levels < 0.05. The overall low abundance of this RNA class in EV (Fig. 2c) hampered reliable quantification of snoRNAs in EV by RT-qPCR (data not shown). Nevertheless, our data are a first indication that EV-associated snoRNA levels change with the activation status of parental cells. Although none of the RNAs in the miscRNA group reached FDR levels < 0.05, analysis by RT-qPCR indicated that the levels of Y3-RNA (Rny3) and an additional member of the Y-RNA family, Y1-RNA (Rny1), significantly differed between LPS and VitD3 conditions (Fig. 5d, e). In cells, the conserved family of Y-RNAs is known to bind to several different proteins that play a role in RNA quality control [75, 76]. Members of the small non-coding Y-RNA family have frequently been detected in EV from multiple different cell types and different body fluids [30–32, 34, 35, 77–81]. We here provide evidence that Y-RNA levels in EV can be regulated by stimuli imposed on the EV-producing cell. The reduced Y-RNA levels in EV from LPS-stimulated DC compared to EV from VitD3-stimulated DC (Fig. 5e) indicate that the presence of Y-RNAs in EV may be indicative for the immune function of the parental cells. Overall, our data indicate that immune cell stimuli cause changes in the EV-associated levels of specific miRNAs, snoRNAs and Y-RNAs.

**Fig. 6:**
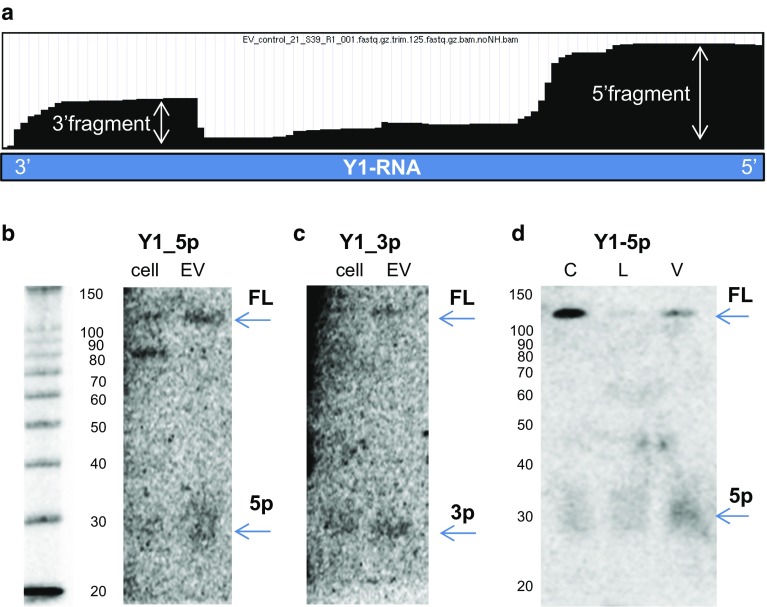
Full-length versus fragmented forms of Y1-RNA in cells and EV validated by Northern blot. **a** Representative coverage plot of Y1-RNA as observed in EV-RNA seq data (sequencing coverage depth 3981) visualized in the UCSC genome browser. **b** RNA isolated from control, LPS, and VitD3-treated cells and their EV were analyzed by Northern blot for the detection of full-length (FL) and 5′fragments (5p) of Y1-RNA using Y1-5p probe. 10 ng of small RNA was loaded per lane. **c** Northern blot detection of full-length (FL) and 3′fragments (3p) of Y1-RNA using Y1-3p probe. 10 ng of small RNA was loaded per lane. **d** Northern blot detection of full-length and 5′fragments of Y1-RNA in EV-RNA from differently stimulated DC (C = control, L = LPS, V = VitD3). 10 ng of small RNA was loaded per lane. Data are representative for *n* = 2 independent experiments

So far, we compared the abundance of specific RNA classes in EV by assessing the total numbers of reads mapping to non-coding RNA genes. However, also the fragmentation profile of EV-associated RNAs may be functionally important and indicative for the status of the EV-producing cell. Fragmented forms of RNA have been described in EV from a wide range of cells and biological fluids [30–32, 34, 35, 77, 78, 81]. Especially, Y-RNA fragments (19–35 nt) have gained attention, because they were found to associate with Argonaute, suggesting scope for gene-regulatory functions [82, 83], and because their presence in plasma has been linked to disease [78, 84]. We examined our data to determine whether reads mapping to Y-RNAs included these fragments in the purified DC-derived EV. Based on RNAseq data, 5′ and 3′ fragments of Y1-RNA were present in EV and such fragments seemed more abundant than the full-length form of this Y-RNA (Fig. 6a). To confirm the full-length or fragmented nature of the Y-RNA molecules, we performed Northern blot analysis. Using probes recognizing the 5′ and 3′ end of Y1-RNA, we found that both 5′ -and 3′ fragments and full-length Y-RNA could be detected in both cellular and EV-RNA (Fig. 6b–d). Strikingly, the relative amount of full-length Y1-RNA detected by Northern blot was much higher than expected based on the sequencing coverage plots. This indicates that RNAseq-based detection of the full-length form of Y1-RNA was highly inefficient. This is likely due to the 5′-triphosphate group of full-length Y-RNAs or their strong secondary structure, which may hamper sequencing adaptor ligation and thereby efficient amplification and sequencing of these RNAs. Interestingly, Northern blot analysis also indicated that the levels of full-length Y1-RNA in LPS-EV were reduced, which was in accordance to the RT-qPCR analysis for full-length Y1 (Fig. 5e), while the levels of fragmented Y1-RNA in EV remained relatively stable (Fig. 6d). These data urge caution in classifying fragmented and full-length forms of Y-RNA based on RNA sequencing data.

**Fig. 7:**
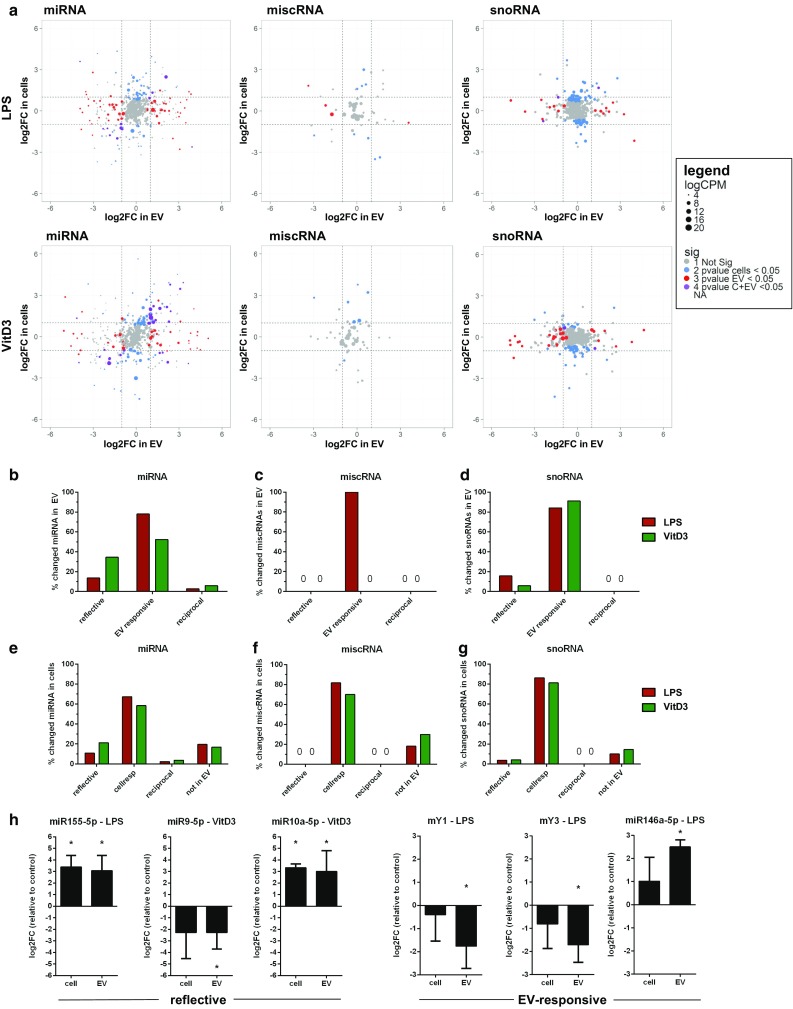
LPS-/VitD3-induced changes in cellular versus EV-associated RNA levels. Cellular and EV-associated RNA from control, LPS, and VitD3 conditions was isolated and analyzed by RNA sequencing. Read counts for individual RNAs were normalized to the total read counts of each RNA class. LPS- or VitD3-induced fold changes and corresponding *p* values were calculated relative to the control condition for cellular and EV-associated RNA. **a** Fold changes in EV-RNAs were plotted against the fold changes in cellular RNA in response to LPS (top graphs) and VitD3 (lower graphs) for miRNA (left), miscRNA (middle), and snoRNA (right). Dashed lines indicate log2FC larger or smaller than 1, so all data points beyond these lines are differentially expressed with log2FC > 1. Colored dots indicate transcripts that changed with non-adjusted *p* values < 0.05 in cells (blue), in EV (red), or in both cells and EV (purple), grey dots indicate unchanged transcripts. Dot size represents the normalized abundance (logCPM) of individual RNAs, mean of *n* = 3 experiments. **b**–**d** Transcripts showing significant changes in stimulated versus control EV (*p* value < 0.05) were selected, and fold changes in EV versus corresponding cells were compared. Indicated are percentages of transcripts categorized as ‘reflective’ (*p* value < 0.05 in EV and cells), ‘EV-responsive’ (*p* value < 0.05 in EV but not in cells), and ‘reciprocal’ (*p* value < 0.05 in cells and EV, but with opposite fold changes). **e**–**g** Analogous to **b**–**d**, transcripts showing significant changes in cells (*p* value < 0.05) were selected, and fold changes in cells were compared with those in EV. Indicated are percentages of transcripts categorized as ‘reflective’, ‘reciprocal’, cell-responsive (*p* value < 0.05 in cells but not in EV), or ‘not in EV’ (transcripts only found in cells). **h** RT-qPCR validation of six genes that were found to be reflective (left panels) or EV-responsive (right panels) on EV-associated RNA and cellular RNA from control, LPS- or VitD3-treated DC. Fold changes in EV and in cells were calculated relative to the control condition in cells or EV. *N* = 4, one-way ANOVA, *p < 0.05

Changes in the RNA content of EV have been suggested to directly reflect changes in the RNA levels of the parental cell. To test this hypothesis, we investigated whether all of the stimulation-induced changes in miRNAs, snoRNAs, and miscRNAs observed in EV reflected changes in the RNA pool of the parental cells and vice versa. After normalization within each RNA class, fold-changes in EV were plotted against the fold changes in cells for miRNA, miscRNA, and snoRNA (Fig. 7a), with dashed lines indicating log2FC of 1 or − 1. Dot size indicates the average (*n* = 3) abundance of a transcript in EV, and dot coloring is used to indicate RNAs that were significantly changed in cells, EV, or both. First, we tested whether changes in EV-associated RNAs reflected stimulation-induced changes in cells. RNAs of which the levels in EV significantly changed (non-adjusted *p* values < 0.05) upon LPS or VitD3-treatment were categorized as ‘reflective RNAs’ (*p* < 0.05 in both cells and EV), ‘EV-responsive RNAs’ (*p* < 0.05 in EV, *p* > 0.05 in cells), or ‘reciprocal RNAs’ (opposite changes in EV and cells with *p* < 0.05) (Supplementary Table 2). Of all RNAs that significantly changed in EV (set to 100%), a minor percentage of the miRNAs and snoRNAs reflected the up/downregulation in cells or were reciprocally regulated, while most of the RNAs changed only in EV, but not (significantly) in cells (Fig. 7b–d). Conversely, we aimed to assess which proportion of LPS- or VitD3-induced changes in cellular RNA was reflected in the EV-RNA content. RNAs that changed in cells upon cell stimulation (non-adjusted *p* values < 0.05) were, therefore, classified as ‘reflective’ (*p* < 0.05 in both cells and EV), ‘cell-responsive’ (*p* < 0.05 in cells, *p* > 0.05 in EV), ‘reciprocal’ (opposite changes in EV and cells with *p* < 0.05), or ‘not in EV’ (*p* < 0.05 in cells, but not detected in EV). Comparable to what we observed in the above analysis of EV-RNA, only a minor percentage of changes in cellular miRNAs and snoRNAs was reflected in EV. The majority of the RNAs that changed in cells did not significantly change in EV (Fig. 7e–g), and 10–20% of the RNAs that changed in cells were not detected in EV. We selected three reflective and three EV-responsive RNAs (Fig. 7h) for validation by RT-qPCR. For the reflective miRNAs miR-155, -9, and -10a, we confirmed that the stimulation-induced fold-change in EV-associated and cellular RNA was highly similar. For the EV-responsive miRNA miR-146a and the two Y-RNAs, significant cell stimulation-induced changes were observed in EV, while cellular levels did not change significantly compared to the control conditions. Importantly, these data indicate that part of the cell stimulation-induced changes in EV-RNA content match the changes observed in cellular RNA, but that cell stimulation also induces changes in RNA levels that are only observed in cells or in EV.

Overall, the results obtained for our primary DC cultures show that the molecular messages enclosed in EV are composed of multiple RNA classes that are specifically shuttled into EV dependent on the activation status of the cell. Moreover, the stimulus-induced changes in the small RNA content of cells and EV only partly overlap.

## Discussion

The data presented here broaden our view on the plasticity of the small RNA content of EV in relation to changes in the activation status of the EV-producing cell. Whereas most of the previous studies focused on the miRNA content of EV, we show that cell status-dependent changes in the RNA composition of EV also extend to other small RNA classes, which may, therefore, also contribute to the specific genetic messages conveyed by EV to recipient cells. The immune cell model which we employed, i.e., DC that are well known for their strong and diverse functional responsiveness to external stimuli, served two purposes. First, the cell system illustrates that diverse stimuli can induce the release of EV with variable levels of multiple RNA types, and that only some of the stimulation-induced changes in EV-associated RNAs reflect changes in the cellular RNA. Second, the data extend our knowledge on the central role of DC in raising and regulating immune responses. The identification of multiple EV-RNA types that are specifically associated with either the immune-activating or immune-suppressing status of DC is the prelude to unraveling the function of these RNAs in EV.

So far, RNA sequencing studies in which multiple RNA classes were identified in EV employed EV isolation methods known to co-isolate extracellular protein–RNA complexes (RNP) that overlap in size with EV, e.g., ultracentrifugation and precipitation-based methods [4, 24, 30, 32, 34, 80, 85, 86]. The results of our present study indicate that up to 55% of total extracellular RNA present in ultracentrifugation pellets of cell culture supernatant can be associated with RNPs. Buoyant density-based separation of EV from RNP, as employed in this study, allowed us to specifically identify RNA species that cells differentially sort into EV upon exogenous stimulation. In RNA sequencing libraries prepared from these highly pure EV, we observed that the percentage of alignment to the mouse genome was lower than in cellular RNA libraries. This is likely caused by the (ultra)low-input quantity of RNA in our EV-sequencing libraries, which amplifies the contribution of lab-derived contaminant RNAs, e.g., from commercial nucleic acid extraction kits or sample cross-contamination [87, 88]. The percentage of alignment may also be dependent on the size range of RNAs selected for sequencing. Whereas, in most published EV-RNA sequencing studies, RNAs in the 20–40 nt size range were sequenced, thereby strongly selecting for microRNAs, we here sequenced RNAs of a much larger size range (20–300 nt). We previously showed that several of these larger and highly modified/structured RNAs, for which the sequencing efficiency may be compromised, are enriched in EV compared to cellular RNA [30], which may have caused the percentages of alignment to be lower in EV compared to cells. For our studies, we selected a commercial adapter-ligation-based method that has been frequently used in EV-RNA sequencing studies [23, 24, 31, 32, 61]. While optimized for miRNA analysis, it should be noted that these library preparation methods show bias in the efficiency with which sequences with base modifications, terminal triphosphate groups, and strong secondary structures are ligated and sequenced [89]. As an example, we here provide Northern blot-based evidence that the frequently reported predominance of Y-RNA fragments over full-length Y-RNA in EV [30, 32, 35, 90] represents a sequencing artefact. We observed a similar discrepancy between sequencing read coverage and Northern blot analysis for tRNAs (Supplementary Fig. 8). While the triphosphate group on the 5′-end [91] or their strong secondary structure may hamper efficient sequencing of full-length Y-RNA, difficulties with sequencing of full-length tRNAs are likely due to the inability of the reverse transcriptase enzyme used during sequencing library preparation to read through the highly modified tRNA structure [92]. This is corroborated by recent sequencing studies deploying reverse transcriptases that are insensitive to secondary RNA structures [93, 94]. Together, these findings urge caution in the interpretation of RNA fragmentation based on RNA sequencing data alone. Moreover, we reduced the effect of sequencing biases on the assessment of quantitative differences in EV-RNA content by determining the fold changes in identical transcripts between different conditions.

Our experimental approach allowed comprehensive analysis of changes in EV-RNA classes induced by diverse immune-relevant stimuli. The most prominent stimulation-induced changes in EV-associated RNA levels were found for miRNA, Y-RNA, and snoRNAs (Fig. 3). Although detected in EV from multiple sources [23, 31, 33–35], the presence of snoRNAs in EV has been largely understudied. Here, we provide evidence that the levels of EV-associated snoRNAs can be regulated by stimuli imposed on the EV-producing cell. Since changes in the cellular levels of snoRNA have been associated with diseases [36, 95, 96], our present data point to EV-associated snoRNAs as potential functional entities in EV and interesting candidate biomarkers indicative of the cellular activation status.

Numerous studies already demonstrated that miRNA levels in EV change upon disease induction or cell stimulation [22–24]. Unique aspects of our present RNAseq study are the use of two different stimuli for comparative analysis of RNA levels in highly purified EV and the parallel assessment of cellular and EV-associated RNA. The enrichment of endotoxin-responsive miRNAs, such as miR-155 and miR-146a [20, 97, 98], that we observed in EV from LPS-stimulated DC concurs with previously published semi-quantitative microarray-based data on the miRNA content of EV from stimulated DC [22, 26]. Both miR-155 and miR-146a are known to be endotoxin-responsive, but miR-155 has an immune-activating role, while miR-146a is involved in dampening of immune responses and could act as a molecular brake on inflammation [99]. It is thought that the coordinated action of these two is important in regulation of immune response [21]. Differences in the miR-155/miR-146a ratios that we observed in LPS-EV and VitD3 EV (Fig. 5b) may, therefore, lead to different effects of these EV on immune activation. In addition, we here demonstrated that a different (i.e., immune downregulatory) type of stimulus imposed on DC led to both a reduction of EV-associated levels of these immune-activating miRNAs and an enrichment in miRNAs implicated in the suppression of immune responses via modulation of multiple pathways. Examples include dampening of TLR signaling by miR-27a and miR-126a [70, 73], and downregulation of (regulators of) PI3K/Akt and NF-κB by miR-378 [100], miR-708 [71] and miR-27b-3p [101], processes that have been implicated in the suppression of pro-inflammatory cytokine release. Such differences in the EV-miRNA content may therefore contribute to immune-activating or immune-suppressive functions of differentially stimulated DC.

Not only immune-suppressive miRNAs but also two types of Y-RNA were found to be specifically excluded from EV released by LPS-stimulated DC (Fig. 5e). This raises the question whether Y-RNAs contribute to immune-related functions of EV. Until now, different types of Y-RNA (mY1 and mY3 in mice and hY1, hY3, hY4, and hY5 in humans) have been detected in EV from a multitude of different cell types and in different body fluids [30–32, 34, 35, 77–81]. The extracellular presence of Y4- and Y5-RNA and fragments thereof has mostly been associated to cancer [32, 81, 84, 102] and coronary artery disease [78, 103]. Immune-related functions of Y-RNAs include their capacity to trigger TLR signaling. Interestingly, Y-RNAs may vary in their specificity for different TLRs, with Y3-RNA triggering predominantly TLR3, while Y1-, Y3-, and Y4-RNA trigger TLR7 [104]. On the contrary, the reduction of Y1- and Y3-RNA levels in EV released by immune-stimulatory DC, shown in this study, suggests an immune downregulatory role for these EV-associated Y-RNAs. In line with this, high levels of Y-RNAs have been reported in immune-suppressive EV released by the parasite *Heligmosomoides polygyrus* and in seminal fluid EV for which immunosuppressive effects have been described [35, 105–107]. Overall, the observed variable presence of Y-RNA in EV from immune cells has raised further interest in unraveling the immune-related function and biomarker potential of these RNAs in EV.

With regard to the biomarker potential of EV-associated RNAs, our data also urge caution in interpreting EV as snapshots of the cell from which they arise. We found that only a subset of changes in EV-RNA reflected changes that occurred at the cellular level. The highest proportion of reflective RNAs was observed within the category of miRNAs and included prominent immune-related RNAs such as miR-155, miR-9, and miR-10-5p (Fig. 7h). For a substantial number of EV-associated miRNAs and both Y-RNA types, however, stimulation-induced changes were observed in EV, but not in cells. This could suggest that EV-levels of reflective RNAs are mainly regulated by transcription, whereas levels of EV-responsive RNAs are mainly determined by shuttling rate. For RNAs known to primarily reside in the nucleus (such as snRNAs and snoRNAs), subtle changes in the cytoplasmic pool of these RNAs may be overshadowed by the much larger nuclear pool. Comparison of EV-associated levels with cytoplasmic instead of total cellular RNA levels may, therefore, provide a better insight in shuttling of these nuclear RNAs into EV. Interestingly, we also observed that many of the stimulation-induced changes in cellular RNA levels were not reflected in the RNA levels of EV released by these cells. This strengthens the hypothesis that cells release EV with selective sets of RNAs into the extracellular milieu. From a biomarker perspective, these data implicate that, although the presence of specific EV-RNAs may correlate with disease, the EV transcriptome is not necessarily predictive for RNA levels in the EV-producing cell.

We observed two classes of RNA, i.e., snRNA and tRNA, of which the EV-associated levels remained stable after differential stimulation of DC. These RNA classes have been commonly detected in small RNAseq studies of EV from multiple cell types and body fluids, but factors impacting their incorporation into EV had until now not been investigated. Although these data need confirmation in different cell types with various external stimuli, our data may be a first indication that tRNAs and snRNAs are constitutive components of EV. Speculatively, these RNAs may be required for basal scaffolding functions in EV biogenesis, or may aid the functional transfer of gene-regulatory RNAs. Based on their stable association with EV, these types of RNAs also have potential to be used as EV-reference RNAs for RT-qPCR normalization, since comparing the EV transcriptome of differentially stimulated cells is complicated by potential differences in the number of EV, RNA yield, and RNA composition. Importantly, the use of well-known stably expressed cellular RNAs is not necessarily valid for normalization of EV qPCR data due to potential differences in RNA shuttling between tested conditions. Therefore, normalization of RT-qPCR data to reference genes which are stable in EV is a reliable way to quantitate differences in individual RNAs. Here, we show how RNA sequencing and RT-qPCR validation can be used to identify candidate EV-reference genes which are constitutively expressed between differently stimulated cells.

In conclusion, this study indicates that external stimuli imposed on cells can lead to changes in the levels of some EV-associated RNA classes, while leaving others unchanged. In our DC-model, two different immune-related cell stimuli led to disparate changes in the type and quantity of miRNAs, snoRNAs, and Y-RNAs in EV. Only part of the changes in RNA contents of EV reflected changes in the cellular RNA, which urges caution in interpreting EV as snapshots of cells. These findings show that differences in multiple structurally distinct RNA classes shape the EV transcriptome. These RNA types may contribute to the functional properties of EV in cellular communication, and may be further explored as candidate EV-RNA-based indicators for the immune status of the EV-producing cell.

## Electronic supplementary material

Below is the link to the electronic supplementary material.

Supplementary material 1 (PDF 2161 kb)

Supplementary material 2 (XLSX 116 kb)

## Abbreviations

BSA: Bovine serum albumin
DC: Dendritic cell
EV: Extracellular vesicles
LPS: Lipopolysaccharide
miRNA/miR: MicroRNA
miscRNA: Miscellaneous RNA
ncRNA: Non-coding RNA
RNP: Ribonucleoprotein (complex)
rRNA: Ribosomal RNA
snRNA: Small nuclear RNA
snoRNA: Small nucleolar RNA
TLR: Toll-like receptor
tRNA: Transfer RNA

## References

[1] Colombo M, Raposo G, Théry C (2014). Biogenesis, secretion, and intercellular interactions of exosomes and other extracellular vesicles. Annu Rev Cell Dev Biol.

[2] van der Grein SG, Nolte-’t Hoen ENM (2014). “Small Talk” in the innate immune system via RNA-containing extracellular vesicles. Front Immunol.

[3] Théry C, Ostrowski M, Segura E (2009). Membrane vesicles as conveyors of immune responses. Nat Rev Immunol.

[4] Mateescu B, Kowal EJK, van Balkom BWM (2017). Obstacles and opportunities in the functional analysis of extracellular vesicle RNA—an ISEV position paper. J Extracell Vesicles.

[5] Van Deun J, Mestdagh P, Agostinis P (2017). EV-TRACK: transparent reporting and centralizing knowledge in extracellular vesicle research. Nat Methods.

[6] Tkach M, Kowal J, The C (2017). Why the need and how to approach the functional diversity of extracellular vesicles. Philos Trans R Soc B Biol Sci.

[7] Camussi G, Deregibus MC, Bruno S (2010). Exosomes/microvesicles as a mechanism of cell-to-cell communication. Kidney Int.

[8] Robbins PD, Morelli AE (2014). Regulation of immune responses by extracellular vesicles. Nat Rev Immunol.

[9] Redzic JS, Balaj L, van der Vos KE, Breakefield XO (2014). Extracellular RNA mediates and marks cancer progression. Semin Cancer Biol.

[10] Revenfeld ALS, Bæk R, Nielsen MH (2014). Diagnostic and prognostic potential of extracellular vesicles in peripheral blood. Clin Ther.

[11] Kahlert C, Melo SA, Protopopov A (2014). Identification of doublestranded genomic dna spanning all chromosomes with mutated KRAS and P53 DNA in the serum exosomes of patients with pancreatic cancer. J Biol Chem.

[12] Raab-Traub N, Dittmer DP (2017). Viral effects on the content and function of extracellular vesicles. Nat Rev Microbiol.

[13] Arroyo JD, Chevillet JR, Kroh EM (2011). Argonaute2 complexes carry a population of circulating microRNAs independent of vesicles in human plasma. Proc Natl Acad Sci USA.

[14] Turchinovich A, Weiz L, Langheinz A, Burwinkel B (2011). Characterization of extracellular circulating microRNA. Nucleic Acids Res.

[15] Vickers KC, Palmisano BT, Shoucri BM (2011). MicroRNAs are transported in plasma and delivered to recipient cells by high-density lipoproteins. Nat Cell Biol.

[16] Van Deun J, Mestdagh P, Sormunen R (2014). The impact of disparate isolation methods for extracellular vesicles on downstream RNA profiling. J Extracell Vesicles.

[17] Lötvall J (2014). Minimal experimental requirements for definition of extracellular vesicles and their functions: a position statement from the. Int Soc Extracell Vesicles..

[18] Vergauwen G, Dhondt B, Van Deun J (2017). Confounding factors of ultrafiltration and protein analysis in extracellular vesicle research. Sci Rep.

[19] O’Connell RM, Taganov KD, Boldin MP (2007). MicroRNA-155 is induced during the macrophage inflammatory response. Proc Natl Acad Sci USA.

[20] Smyth LA, Boardman DA, Tung SL (2015). MicroRNAs affect dendritic cell function and phenotype. Immunology.

[21] Mehta A, Baltimore D (2016). MicroRNAs as regulatory elements in immune system logic. Nat Rev Immunol.

[22] Montecalvo A, Larregina AT, Shufesky WJ (2012). Mechanism of transfer of functional microRNAs between mouse dendritic cells via exosomes. Blood.

[23] Bellingham SA, Coleman BM, Hill AF (2012). Small RNA deep sequencing reveals a distinct miRNA signature released in exosomes from prion-infected neuronal cells. Nucleic Acids Res.

[24] Cha DJ, Franklin JL, Dou Y (2015). KRAS-dependent sorting of miRNA to exosomes. Elife.

[25] Valadi H, Ekström K, Bossios A (2007). Exosome-mediated transfer of mRNAs and microRNAs is a novel mechanism of genetic exchange between cells. Nat Cell Biol.

[26] Alexander M, Hu R, Runtsch MC (2015). Exosome-delivered microRNAs modulate the inflammatory response to endotoxin. Nat Commun.

[27] Taylor DD, Gercel-Taylor C (2008). MicroRNA signatures of tumor-derived exosomes as diagnostic biomarkers of ovarian cancer. Gynecol Oncol.

[28] Murata K, Yoshitomi H, Tanida S (2010). Plasma and synovial fluid microRNAs as potential biomarkers of rheumatoid arthritis and osteoarthritis. Arthritis Res Ther.

[29] Cheng L, Doecke JD, Sharples RA (2014). Prognostic serum miRNA biomarkers associated with Alzheimer’s disease shows concordance with neuropsychological and neuroimaging assessment. Mol Psychiatry.

[30] Nolte-’t Hoen ENM, Buermans HPJ, Waasdorp M (2012). Deep sequencing of RNA from immune cell-derived vesicles uncovers the selective incorporation of small non-coding RNA biotypes with potential regulatory functions. Nucleic Acids Res.

[31] van Balkom BWM, Eisele AS, Pegtel DM (2015). Quantitative and qualitative analysis of small RNAs in human endothelial cells and exosomes provides insights into localized RNA processing, degradation and sorting. J Extracell Vesicles.

[32] Tosar JP, Gambaro F, Sanguinetti J (2015). Assessment of small RNA sorting into different extracellular fractions revealed by high-throughput sequencing of breast cell lines. Nucleic Acids Res.

[33] Yeri A, Courtright A, Reiman R (2017). Total extracellular small RNA profiles from plasma, saliva, and urine of healthy subjects. Sci Rep.

[34] Lunavat TR, Cheng L, Kim D-K (2015). Small RNA deep sequencing discriminates subsets of extracellular vesicles released by melanoma cells—evidence of unique microRNA cargos. RNA Biol.

[35] Vojtech L, Woo S, Hughes S (2014). Exosomes in human semen carry a distinctive repertoire of small non-coding RNAs with potential regulatory functions. Nucleic Acids Res.

[36] Youssef OA, Safran SA, Nakamura T (2015). Potential role for snoRNAs in PKR activation during metabolic stress. Proc Natl Acad Sci USA.

[37] Michel CI, Holley CL, Scruggs BS (2011). Small nucleolar RNAs U32a, U33 and U35a are critical mediators of metabolic stress. Cell Metab.

[38] Stepanov GA, Filippova JA, Komissarov AB (2015). Regulatory role of small nucleolar RNAs in human diseases. Biomed Res Int.

[39] Haussecker D, Huang Y, Lau A (2010). Human tRNA-derived small RNAs in the global regulation of RNA silencing. RNA.

[40] Ninomiya S, Kawano M, Abe T (2015). Potential small guide RNAs for tRNase ZL from human plasma, peripheral blood mononuclear cells, and cultured cell lines. PLoS One.

[41] Nabet BY, Qiu Y, Shabason JE (2017). Exosome RNA unshielding couples stromal activation to pattern recognition receptor signaling in cancer. Cell.

[42] Tkach M, Thery C (2016). Communication by extracellular vesicles: where we are and where to go. Cell.

[43] Zitvogel L, Regnault A, Lozier A (1998). Eradication of established murine tumors using a novel cell-free vaccine: dendritic cell-derived exosomes. Nat Med.

[44] Pitt JM, André F, Amigorena S (2016). Dendritic cell-derived exosomes for cancer therapy. J Clin Investig.

[45] Wu T, Qi Y, Zhang D (2017). Bone marrow dendritic cells derived microvesicles for combinational immunochemotherapy against tumor. Adv Funct Mater.

[46] Tao SC, Guo SC, Zhang CQ (2018). Modularized extracellular vesicles: the dawn of prospective personalized and precision medicine. Adv Sci.

[47] Raker VK, Domogalla MP, Steinbrink K (2015). Tolerogenic dendritic cells for regulatory T cell induction in man. Front Immunol.

[48] Lutz MB, Kukutsch N, Ogilvie AL (1999). An advanced culture method for generating large quantities of highly pure dendritic cells from mouse bone marrow. J Immunol Methods.

[49] van der Vlist EJ, Nolte-’t Hoen ENM, Stoorvogel W (2012). Fluorescent labeling of nano-sized vesicles released by cells and subsequent quantitative and qualitative analysis by high-resolution flow cytometry. Nat Protoc.

[50] Robinson MD, McCarthy DJ, Smyth GK (2009). edgeR: a bioconductor package for differential expression analysis of digital gene expression data. Bioinformatics.

[51] Robinson JT, Thorvaldsdóttir H, Winckler W (2011). Integrative genomics viewer. Nat Biotechnol.

[52] Nolte-’t Hoen ENM, van der Vlist EJ, Aalberts M (2012). Quantitative and qualitative flow cytometric analysis of nanosized cell-derived membrane vesicles. Nanomedicine.

[53] Groot Kormelink T, Arkesteijn GJA, Nauwelaers FA (2016). Prerequisites for the analysis and sorting of extracellular vesicle subpopulations by high-resolution flow cytometry. Cytom Part A.

[54] Libri V, Helwak A, Miesen P (2012). Murine cytomegalovirus encodes a miR-27 inhibitor disguised as a target. Proc Natl Acad Sci USA.

[55] Pall GS, Hamilton AJ (2008). Improved northern blot method for enhanced detection of small RNA. Nat Protoc.

[56] Piemonti L, Monti P, Sironi M (2000). Vitamin D3 affects differentiation, maturation, and function of human monocyte-derived dendritic cells. J Immunol.

[57] Unger WWJ, Laban S, Kleijwegt FS (2009). Induction of Treg by monocyte-derived DC modulated by vitamin D3 or dexamethasone: differential role for PD-L1. Eur J Immunol.

[58] Penna G, Adorini L (2000). 1,25-dihydroxyvitamin D3 inhibits differentiation, maturation, activation, and survival of dendritic cells leading to impaired alloreactive T cell activation. J Immunol.

[59] Thery C, Boussac M, Veron P (2001). Proteomic analysis of dendritic cell-derived exosomes: a secreted subcellular compartment distinct from apoptotic vesicles. J Immunol.

[60] ’t Hoen PAC, Friedländer MR, Almlöf J (2013). Reproducibility of high-throughput mRNA and small RNA sequencing across laboratories. Nat Biotechnol.

[61] Koppers-Lalic D, Hackenberg M, Bijnsdorp IV (2014). Nontemplated nucleotide additions distinguish the small RNA composition in cells from exosomes. Cell Rep.

[62] Dueck A, Eichner A, Sixt M, Meister G (2014). A miR-155-dependent microRNA hierarchy in dendritic cell maturation and macrophage activation. FEBS Lett.

[63] Ma Z-X, Tan X, Shen Y (2015). MicroRNA expression profile of mature dendritic cell in chronic rhinosinusitis. Inflamm Res.

[64] Wu W, He C, Liu C (2015). miR-10a inhibits dendritic cell activation and Th1/Th17 cell immune responses in IBD. Gut.

[65] Park H, Huang X, Lu C (2015). MicroRNA-146a and microRNA-146b regulate human dendritic cell apoptosis and cytokine production by targeting TRAF6 and IRAK1 proteins. J Biol Chem.

[66] Bazzoni F, Rossato M, Fabbri M (2009). Induction and regulatory function of miR-9 in human monocytes and neutrophils exposed to proinflammatory signals. Proc Natl Acad Sci USA.

[67] Zhou H, Xiao J, Wu N (2015). MicroRNA-223 regulates the differentiation and function of intestinal dendritic cells and macrophages by targeting C/EBP-beta. Cell Rep.

[68] Pedersen AW, Holmstrøm K, Jensen SS (2009). Phenotypic and functional markers for 1alpha,25-dihydroxyvitamin D(3)-modified regulatory dendritic cells. Clin Exp Immunol.

[69] Stumpfova Z, Hezova R, Meli AC (2014). MicroRNA profiling of activated and tolerogenic human dendritic cells. Mediat Inflamm.

[70] Agudo J, Ruzo A, Tung N (2013). The miR-126-VEGFR2 axis controls the innate response to pathogen-associated nucleic acids. Nat Immunol.

[71] Baer C, Oakes CC, Ruppert AS (2015). Epigenetic silencing of miR-708 enhances NF-kB signaling in chronic lymphocytic leukemia. Int J Cancer.

[72] Lin J, Xia J, Tu CZ (2017). H9N2 avian influenza virus protein PB1 enhances the immune responses of bone marrow-derived dendritic cells by down-regulating miR375. Front Microbiol.

[73] Lv Y, Ou-yang A, Fu L (2017). MicroRNA-27a negatively modulates the inflammatory response in lipopolysaccharide-stimulated microglia by targeting TLR4 and IRAK4. Cell Mol Neurobiol.

[74] Vandesompele J, De Preter K, Pattyn F (2002). Accurate normalization of real-time quantitative RT-PCR data by geometric averaging of multiple internal control genes. Genome Biol.

[75] Stein AJ, Fuchs G, Fu C (2005). Structural insights into RNA quality control: the Ro autoantigen binds misfolded RNAs via its central cavity. Cell.

[76] Chen X, Smith JD, Shi H (2003). The Ro autoantigen binds misfolded U2 small nuclear RNAs and assists mammalian cell survival after UV irradiation. Curr Biol.

[77] Dhahbi JM, Spindler SR, Atamna H (2013). 5′-YRNA fragments derived by processing of transcripts from specific YRNA genes and pseudogenes are abundant in human serum and plasma. Physiol Genom.

[78] Repetto E, Lichtenstein L, Hizir Z (2015). RNY-derived small RNAs as a signature of coronary artery disease. BMC Med.

[79] Kaudewitz D, Skroblin P, Bender LH (2015). Association of microRNAs and YRNAs with platelet function. Circ Res.

[80] Cambier L, de Couto G, Ibrahim A (2017). Y RNA fragment in extracellular vesicles confers cardioprotection via modulation of IL-10 expression and secretion. EMBO Mol Med.

[81] Haderk F, Schulz R, Iskar M (2017). Tumor-derived exosomes modulate PD-L1 expression in monocytes. Sci Immunol.

[82] Meiri E, Levy A, Benjamin H (2010). Discovery of microRNAs and other small RNAs in solid tumors. Nucleic Acids Res.

[83] Thomson DW, Pillman KA, Anderson ML (2015). Assessing the gene regulatory properties of argonaute-bound small RNAs of diverse genomic origin. Nucleic Acids Res.

[84] Dhahbi JM, Spindler SR, Atamna H (2014). Deep sequencing of serum small RNAs identifies patterns of 5′ tRNA half and YRNA fragment expression associated with breast cancer. Biomark Cancer.

[85] Li CCY, Eaton SA, Young PE (2013). Glioma microvesicles carry selectively packaged coding and non-coding RNAs which alter gene expression in recipient cells. RNA Biol.

[86] Fiskaa T, Knutsen E, Nikolaisen MA (2016). Distinct small rna signatures in extracellular vesicles derived from breast cancer cell lines. PLoS One.

[87] Tosar JP, Rovira C, Naya H, Cayota A (2014). Mining of public sequencing databases supports a non-dietary origin for putative foreign miRNAs: underestimated effects of contamination in NGS. RNA.

[88] Tosar JP, Cayota A, Eitan E (2017). Ribonucleic artefacts: are some extracellular RNA discoveries driven by cell culture medium components?. J Extracell Vesicles.

[89] Raabe CA, Tang T-H, Brosius J, Rozhdestvensky TS (2014). Biases in small RNA deep sequencing data. Nucleic Acids Res.

[90] Wei Z, Batagov AO, Schinelli S (2017). Coding and noncoding landscape of extracellular RNA released by human glioma stem cells. Nat Commun.

[91] Hendrick JP, Wolin SL, Rinke J (1981). Ro small cytoplasmic ribonucleoproteins are a subclass of La ribonucleoproteins: further characterization of the Ro and La small ribonucleoproteins from uninfected mammalian cells. Mol Cell Biol.

[92] Motorin Y, Muller S, Behn-Ansmant I, Branlant C (2007). Identification of modified residues in RNAs by reverse transcription-based methods. Methods enzymol.

[93] Qin Y, Yao J, Wu DC (2015). High-throughput sequencing of human plasma RNA by using thermostable group II intron reverse transcriptases. RNA.

[94] Shurtleff MJ, Yao J, Qin Y (2017). Broad role for YBX1 in defining the small noncoding RNA composition of exosomes. Proc Natl Acad Sci.

[95] Gee HE, Buffa FM, Camps C (2011). The small-nucleolar RNAs commonly used for microRNA normalisation correlate with tumor pathology and prognosis. Br J Cancer.

[96] Martens-Uzunova ES, Olvedy M, Jenster G (2013). Beyond microRNA—novel RNAs derived from small non-coding RNA and their implication in cancer. Cancer Lett.

[97] Baltimore D, Boldin MP, O’Connell RM (2008). MicroRNAs: new regulators of immune cell development and function. Nat Immunol.

[98] O’Connell RM, Kahn D, Gibson WSJ (2010). MicroRNA-155 promotes autoimmune inflammation by enhancing inflammatory T cell development. Immunity.

[99] Boldin MP, Taganov KD, Rao DS (2011). miR-146a is a significant brake on autoimmunity, myeloproliferation, and cancer in mice. J Exp Med.

[100] Ruckerl D, Jenkins SJ, Laqtom NN (2012). Induction of IL-4R alpha-dependent microRNAs identifies PI3K/Akt signaling as essential for IL-4-driven murine macrophage proliferation in vivo. Blood.

[101] Lee J-J, Drakaki A, Illiopoulos D, Struhl K (2012). MiR-27b targets PPARy to inhibit growth, tumor progression, and the inflammatory response in neuroblastoma cells. Oncogene.

[102] Chakrabortty SK, Prakash A, Nechooshtan GAL (2015). Extracellular vesicle-mediated transfer of processed and functional RNY5 RNA. RNA.

[103] Hizir Z, Bottini S, Grandjean V (2017). RNY (YRNA)-derived small RNAs regulate cell death and inflammation in monocytes/macrophages. Cell Death Dis.

[104] Greidinger EL, Zang Y, Martinez L (2007). Differential tissue targeting of autoimmunity manifestations by autoantigen-associated Y RNAs. Arthritis Rheumatol.

[105] Skibinski G, Kelly R, Harkiss D, James K (1992). Immunosuppression by human seminal plasma—extracellular organelles (prostasomes) modulate activity of phagocytic cells. Am J Reprod Immunol.

[106] Tarazona R, Delgado E, Guarnizo MC (2011). Human prostasomes express CD48 and interfere with NK cell function. Immunobiology.

[107] Buck AH, Coakley G, Simbari F (2014). Exosomes secreted by nematode parasites transfer small RNAs to mammalian cells and modulate innate immunity. Nat Commun.

