# Supplementary material for: Immune stimuli shape the small non-coding transcriptome of extracellular vesicles released by dendritic cells
Source: Cell Mol Life Sci. 2018 May 28;75(20):3857–75. doi: 10.1007/s00018-018-2842-8 (PMC6154026; doi:10.1007/s00018-018-2842-8)

Supplementary Figures & Table

Supplementary Figure 1. Generation of immune-activating and immune-suppressive DC subsets by treatment with LPS or VitD3

DCs were left untreated (control), treated with LPS to induce immune-stimulatory DC, or treated with 1α,25-$(OH)_2$-VitaminD_3 to induce immune-suppressive DC. **A – E)** Cell surface expression of CD11c, MHCII, CD40, PD-L1, and CD86 on control, LPS- or VitD3-treated DC was determined by flow-cytometry at the start of the EV-production phase. **F)** The ratio between PD-L1 and CD86 was determined as a measure for tolerogenicity. Data are expressed as isotype-corrected mean fluorescence intensity values, normalized against expression in control (untreated) DC cultures. Indicated are mean +/- s.d. fold induction of n=8 independent experiments, one-way ANOVA with Dunnet's two-sided post-hoc test, *$p < 0.05$

Supplementary Figure 2. Isolation and characterization of dendritic cell-derived EV and their RNA content.

A) Schematic representation of the differential centrifugation protocol used to generate highly purified EV from cell culture supernatant. Large contaminants such as cell debris and apoptotic bodies are depleted during the low-speed centrifugation steps, after which EV are pelleted from the cleared supernatant by ultracentrifugation. Sucrose density gradient centrifugation was used to separate low density EV from high density ribonucleoprotein complexes (RNP). **B)** Electron microscopy images of EV isolated from 1.12 – 1.18 g/ml density fractions (top panels) and from 1.28 g/ml RNP-containing fractions (bottom panels). Scale bars represent 100 nm (left panels) and 500 nm (right panels) **C)** High-resolution flow cytometric analysis of purified PKH67-labeled EV (1.14 g/ml) from differentially stimulated DC. Representative dot plots indicate no apparent differences in FSC/SSC light scatter profiles (top panels) or fluorescence intensity (PKH, bottom panels) between EV in the indicated conditions. 10,000 events are plotted per graph. **D)** Purified EV (1.12-1.18 g/ml) from differentially stimulated DCs were analyzed by Nanoparticle Tracking Analysis. Indicated are the size distributions of EV in the different DC conditions and relative differences in numbers of EV released by these DC (bar graph). **E)** Lysates of control (C), LPS-treated (L), or VitD3-treated (V) cells (left) and purified EV released under these conditions (right) were analyzed for the presence of CD63, Galectin-3, MHCII, CD9 and beta-actin by Western blotting. For cell lysates, an equal amount of protein was loaded for each condition. For comparison of EV protein composition, equal numbers of EV released in each condition were loaded, as based on high-resolution flow cytometric and NTA-based analysis. Beta actin was not detectable in EV, even at prolonged exposure times. **F)**

Representative Bioanalyzer Pico 6000 electropherograms of RNA isolated from floating EV and non-floating RNP, showing differences in RNA size profiles. **G)** Bioanalyzer-based quantification of RNA yields from EV and RNP. Indicated are the percentages of total extracellular RNA associated to either EV or RNP (mean +/- s.d. of n=3 independent experiments).

Supplementary Figure 3. Ratiometric pooling of size fractions improves RNA sequencing-based coverage of 100-300 nt mid-size RNAs

A) Representative IGV Genome Viewer plots of RNAseq read coverage of Vault-RNA after ratiometric pooling (top) or under standard library preparation conditions (bottom). **B)** Reads were counted for the 3' fragment and the middle part of Vault RNA, as indicated. These values were divided by the total number of reads detected for Vault RNA and compared for ratiometric versus standard conditions.

Supplementary Table 1. Library size, quality control and alignment percentages of all EV and cellular RNA libraries

Overview of the RNAseq libraries prepared for cellular and EV RNA in this study, the corresponding indices, read depth, and quality control parameters.

Supplementary Figure 4. Reproducibility of biological replicate libraries for cells and EV

Cellular and EV-associated RNA from control, LPS, and VitD3 conditions was isolated and analyzed by RNA sequencing. The RPM for all biological replicate libraries were plotted against one another. Pearson coefficients are indicated in the plots.

Supplementary Figure 5. RNA class distribution in cells and EV upon immune stimulation

Class distribution in RNA from differently stimulated DCs **(A)** and corresponding EV **(B)** was analyzed by RNA sequencing. For each of the RNAseq libraries, total read counts for each RNA class were expressed as a percentage of the total library size. Average percentage of reads for each RNA class (of n=3 replicate libraries) is plotted for each of the treatments. No major changes in RNA class distribution can be observed as a result of stimuli imposed on the DCs.

Supplementary Figure 6. EV from LPS- and VitD3-stimulated DC display stable levels of abundant tRNAs

EV-associated RNA from control, LPS, and VitD3 conditions was isolated and analyzed by RNA sequencing. Read counts for individual RNAs were normalized to the total read counts of each RNA class. **A)** Read counts for the top-6 most abundant tRNAs, constituting 97% of tRNA reads in EV-RNA, are expressed as percentage of the total tRNA read count in EV. **B)** Data from (A) are expressed as fold-change in the indicated tRNA levels of VitD3- or LPS-EV relative to control-EV (mean +/- s.d. of n=3 experiments, one-way ANOVA, * $p < 0.05$).

Supplementary Figure 7. Validation of reference RNAs in EV for normalization of qPCR data

A) EV-RNA sequencing data were used to validate the stable presence of indicated candidate reference small RNAs in EV from differentially stimulated DC. Indicated are the LPS-and VitD3-induced fold changes in EV relative to the control EV of n=3 independent experiments. **B)** RT-qPCR validation of candidate reference RNAs identified in (A). RT-qPCR reactions were performed with equal RNA input from control- , LPS-, and VitD3-EV. Indicated are individual and mean Cq values of snord65, snord68, U1 and U6 from 3 – 4 independent experiments. The geometric mean of these four reference genes, as shown on the right side of (B) was used as normalization value for EV qPCR data.

Supplementary Figure 8. Northern blot analysis to validate the ratio of full-length versus fragmented forms of tRNA-Glu-CTC and tRNA-Gly-GCC in cells and EV

A) RNA isolated from control, LPS, and VitD3-treated cells and their EVs were analysed by Northern blot for detection of tRNA-Glu-CTC in using 5'tRNA-Glu probe **B)** The blot in (A) was stripped and reprobed for tRNA-Gly-GCC using 5'tRNA-Gly probe. Full length tRNA and tRNA fragments (tRF) are indicated with arrows. 10 ng of cellular smallRNA and 1 ng of EV-smallRNA were loaded per lane. Data are representative for n=2 independent experiments. **C)** Representative coverage plot of tRNA-Glu-CTC as observed in EV RNA seq data (sequencing coverage depth 463,870) visualized in the UCSC Genome Browser.

Supplementary Figure 1

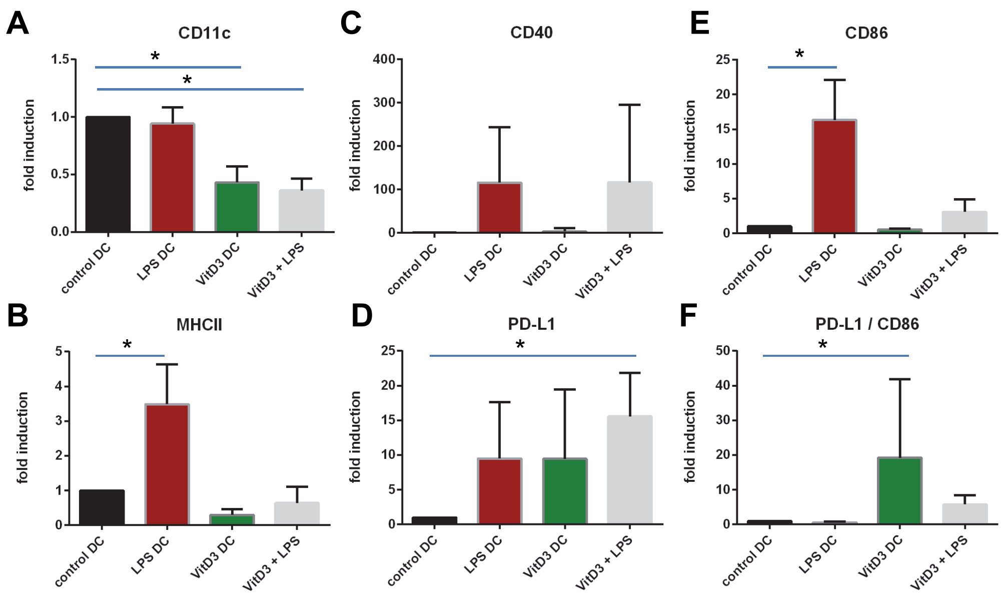

Supplementary Figure 2

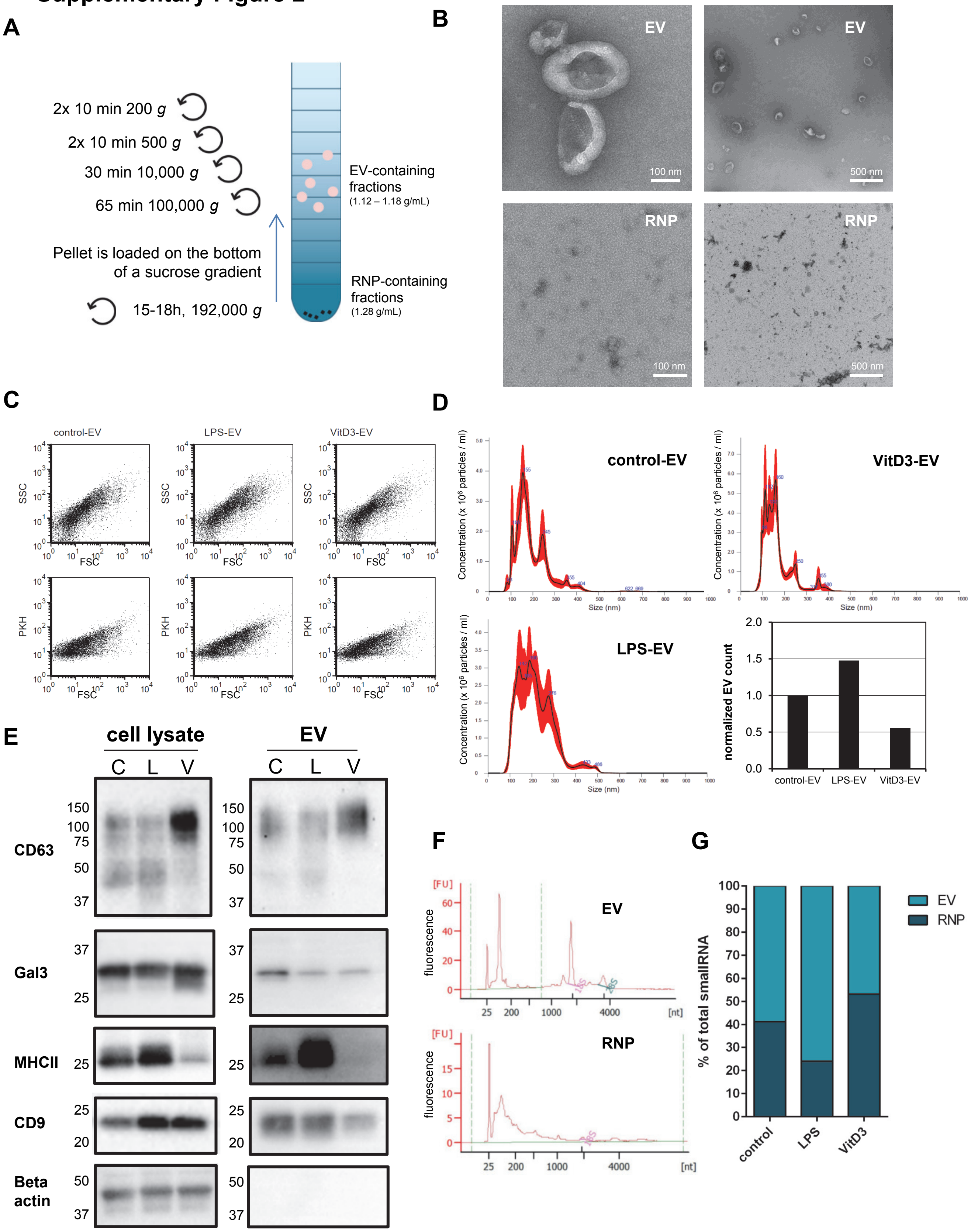

Supplementary Figure 3

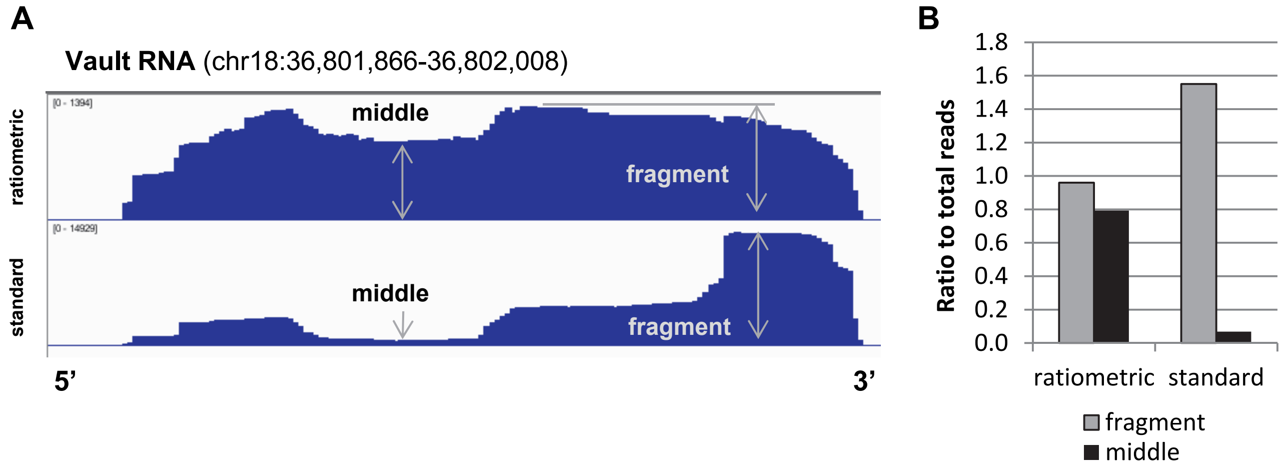

Supplementary Table 1

Sample	index	# reads	# reads pass filter	% Pass	% Alignment
cell_control_S4	11	24,184,761	23,206,725	96%	84.50%
cell_control_S7	14	16,891,584	15,848,861	94%	76.80%
cell_control_S36	18	23,827,849	21,077,194	88%	75.12%
cell_LPS_S5	12	19,526,758	18,187,862	93%	83.50%
cell_LPS_S8	15	14,816,903	13,494,221	91%	80.80%
cell_LPS_S37	19	48,426,527	45,972,567	95%	85.35%
cell_VitD3_S6	13	15,236,904	14,543,618	95%	82.90%
cell_VitD3_S9	16	13,004,444	12,242,191	94%	76.20%
cell_VitD3_S38	20	38,148,808	33,109,203	87%	74.31%
EV_cont_S10	17	15,138,107	11,336,408	75%	33.60%
EV_cont_S13	20	18,222,150	8,168,987	45%	28.30%
EV_control_S39	21	22,687,712	15,110,524	67%	36.84%
EV_LPS_S11	18	16,111,933	12,326,349	77%	29.30%
EV_LPS_S14	21	11,150,431	4,269,979	38%	29.20%
EV_LPS_S40	22	21,884,586	13,320,228	61%	30.99%
EV_VitD3_S12	19	16,296,162	9,957,537	61%	27.30%
EV_VitD3_S15	22	13,273,020	7,856,779	59%	26.90%
EV_VitD3_S41	23	15,662,964	7,968,405	51%	20.31%

Supplementary Figure 4

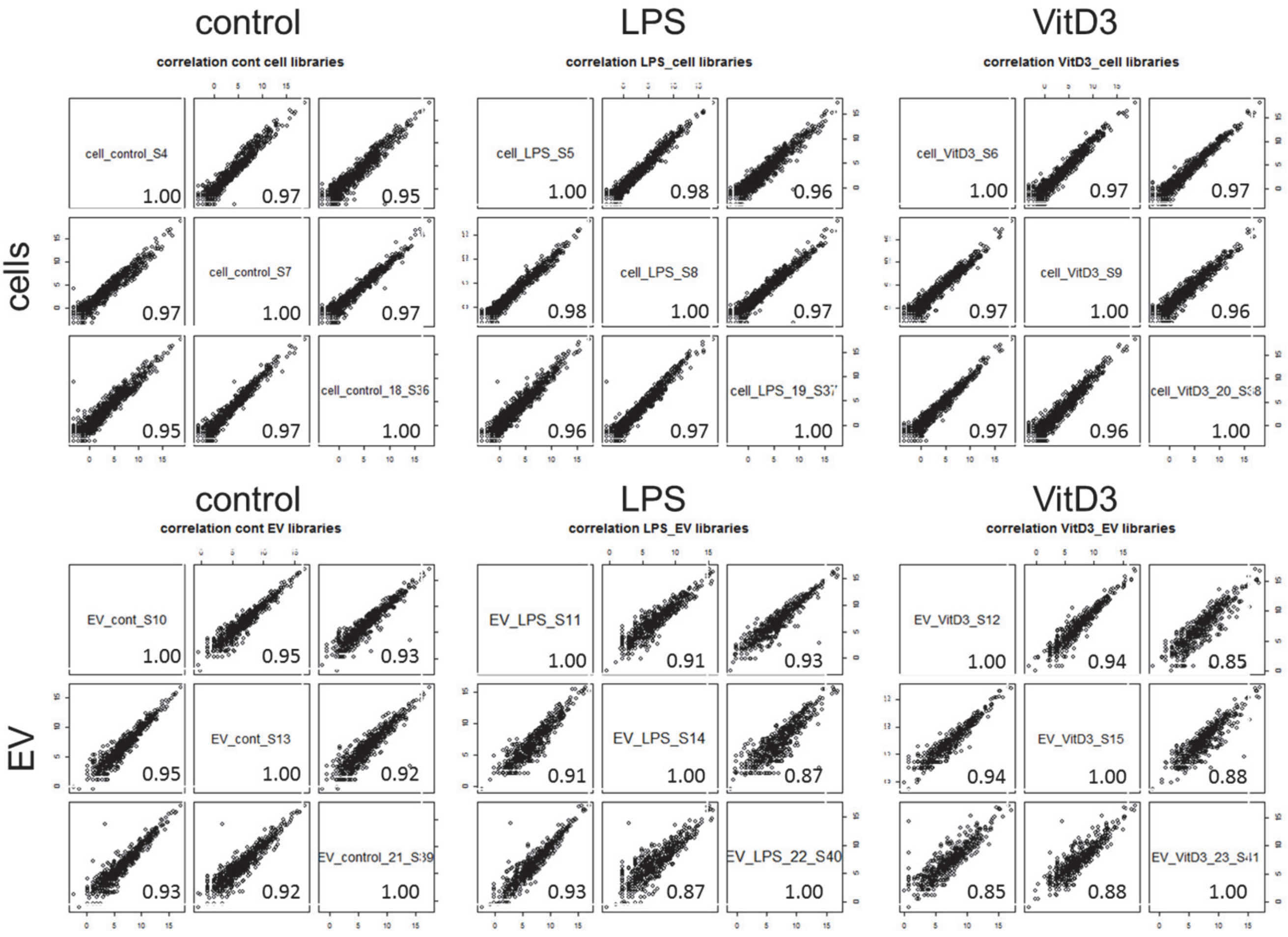

Supplementary Figure 5

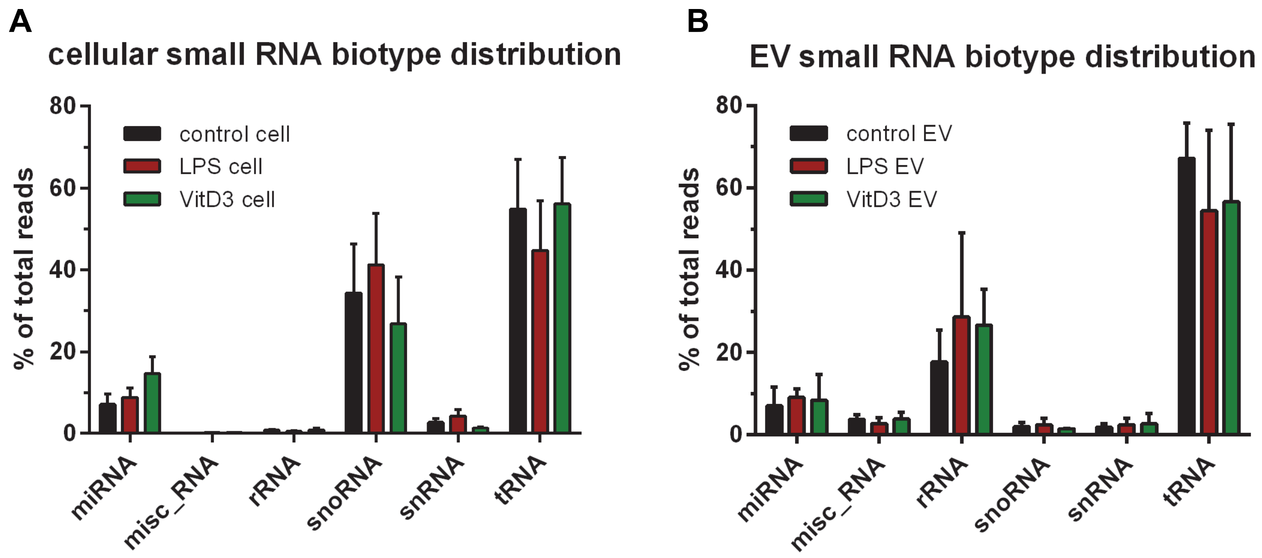

Supplementary Figure 6

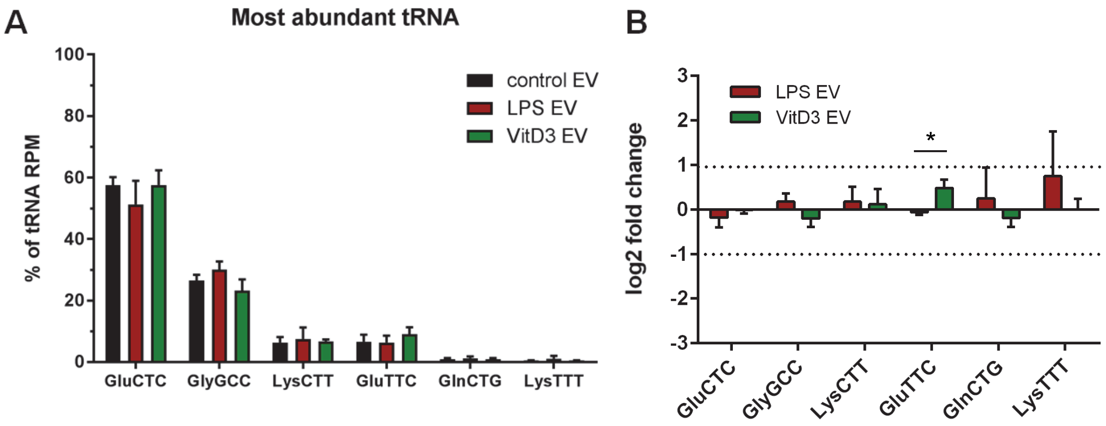

Supplementary Figure 7

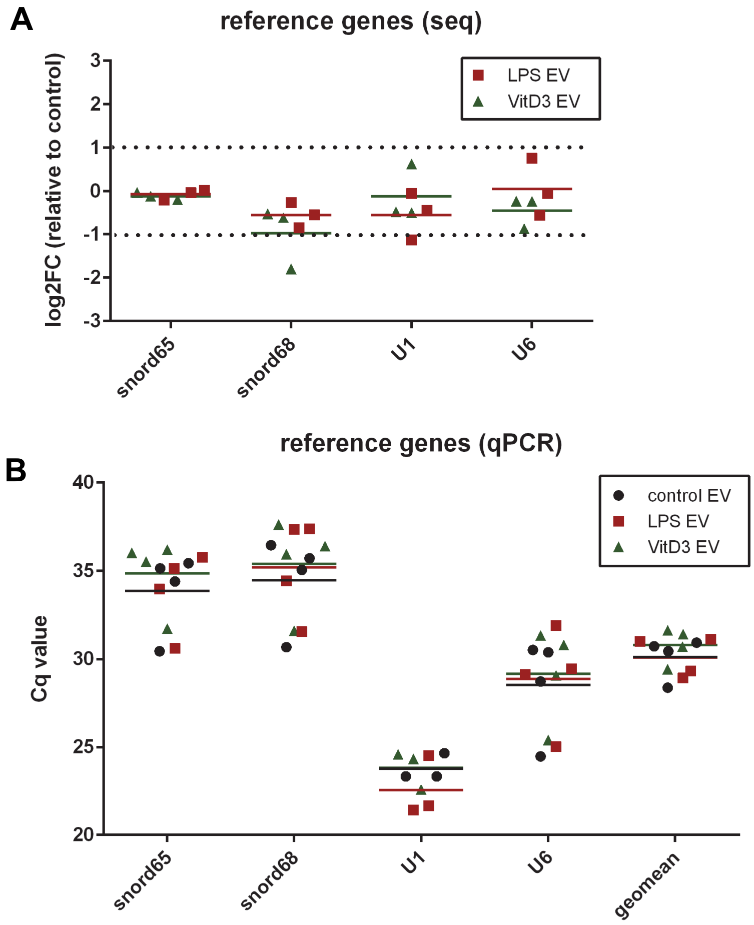

Supplementary Figure 8

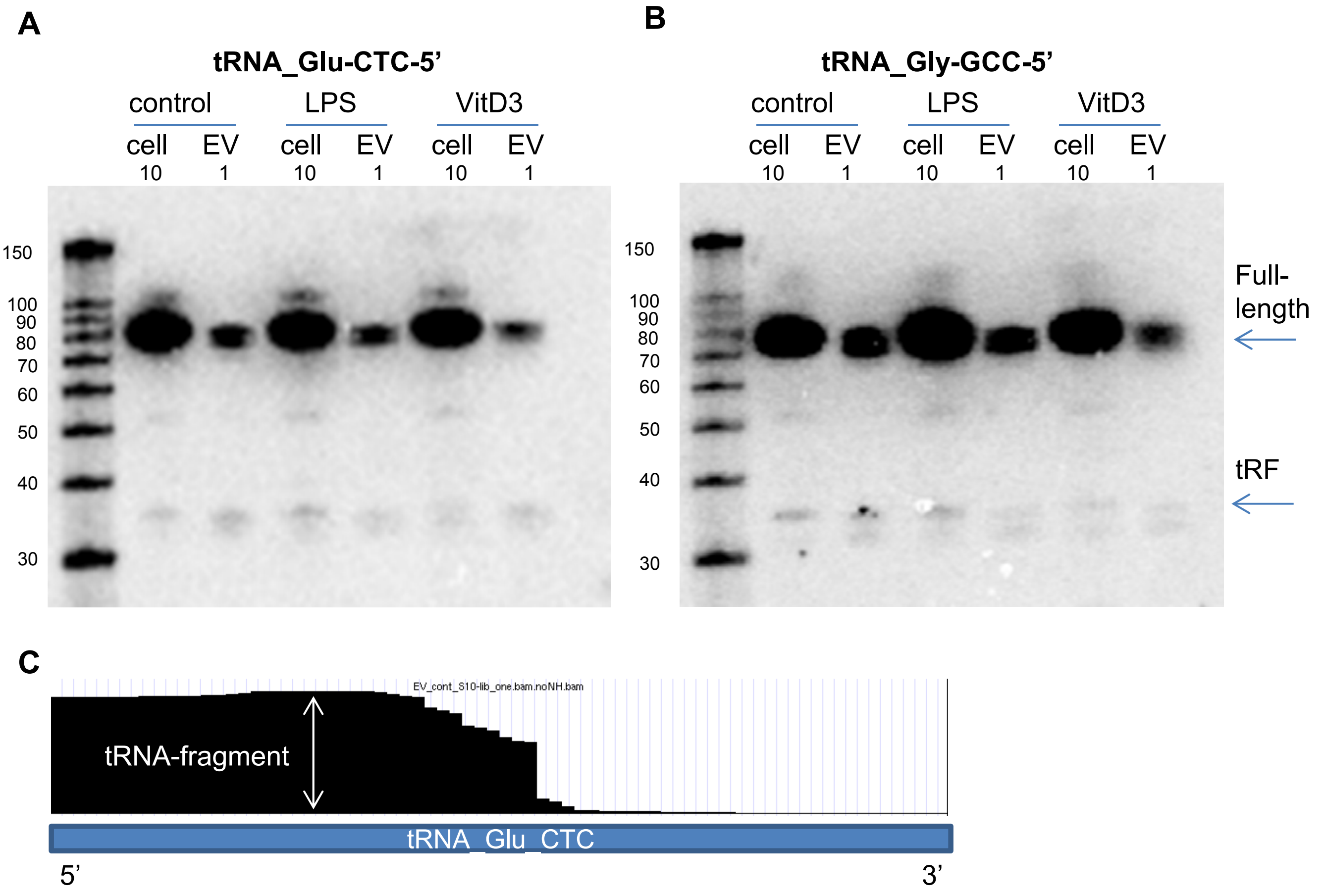

Supplement: Supplementary file 1 — Supplementary material 1 (PDF 2161 kb) [file 18_2018_2842_MOESM1_ESM.pdf]
